# Whole-genome characterization of *Rosa chinensis* AP2/ERF transcription factors and analysis of negative regulator *RcDREB2B* in Arabidopsis

**DOI:** 10.1186/s12864-021-07396-6

**Published:** 2021-01-28

**Authors:** Wei Li, Ziwen Geng, Cuiping Zhang, Kuiling Wang, Xinqiang Jiang

**Affiliations:** College of Landscape Architecture and Forestry, Qingdao Agricultural University, Qingdao, 266000 China

**Keywords:** Rose, AP2/ERF, Transcription factor, *RcDREB2B*, Drought

## Abstract

**Background:**

Rose (*Rosa chinensis*) is a traditional famous flower with valuable ornamental characteristics. However, drought stress restricts its growth and development, leading to an abnormal phenotype. One of the main transcription factor (TF) protein groups in the plant kingdom are the APETALA2/ethylene-responsive factor (AP2/ERF) proteins and are potentially involved in the growth and stress responses of various plants.

**Results:**

Our investigation mainly focused on exploring the genome of rose and thereby we discovered 135 apparent AP2/ERF TFs. Phylogenic analyses revealed that *RcAP2/ERF* genes are categorized into DREB, Soloist, AP2, and ERF subfamilies, and are further classified these into 17 groups, with the same as *Malus domestica* and *Arabidopsis thaliana*. The analysis of the gene structure revealed that the introns ranged from 0 to 9 in number. Pattern examination demonstrated that the *RcAP2/ERF* predominantly consists of typical AP2 domains, of which the 2nd motif is the most ubiquitous. Distributions of *cis*-acting elements indicated that members of the AP2/ERF family are frequently involved in growth and development, phytohormone and stress response in rose species. Also, the distribution mapping of the rose chromosomes indicated that AP2/ERF class genes are dispersed among all seven chromosomes. Additionally, we isolated a novel DREB A2 subgroup gene and named it *RcDREB2B*. Subsequently, the *RcDREB2B* transcript accumulation was repressed under the mild and severe drought stress in the root samples of rose. RcDREB2B was targeted to the nucleus and exhibited transactivation in yeast cells. The overexpression of *RcDREB2B* was found to promote sensitivity to a higher salt concentration, ABA, and PEG at the germination and post-germination stages. Twelve putative osmotic and ABA-related genes were impaired in *RcDREB2B*-overexpressing plants.

**Conclusions:**

The results provide comprehensive information regarding the gene structure, phylogenic, and distribution of the rose AP2/ERF family and bring insight into the complex transcriptional gene regulation of *RcAP2/ERF*. Findings in this study would also contribute to further understanding of the *RcDREB2B* gene in rose.

**Supplementary Information:**

The online version contains supplementary material available at 10.1186/s12864-021-07396-6.

## Background

Transcription factors (TFs) mediate plant developmental and growth processes. Besides, they also perform vital roles in transmitting stimulatory or inhibitory signals [1, 2]. APETALA2/ethylene-responsive factor (AP2/ERF) is one of the major TF superfamilies in plants. They possess either single or double AP2 DNA-binding domains that have a sequence homology of approximately 70 amino acid residues [3]. According to AP2 domain numbers and sequence similarity, AP2/ERF is classified into three subgroups, namely ERF, RAV, and AP2 gene subfamilies [3]. Whereas, the ERF subfamily contains a single AP2 domain while the AP2 subfamily has two. Both an AP2 domain and a B3 DNA-binding domain are present in the RAV subgroup [4]. Also, based on the variations in binding promoter sequences of the ERF family can be split into DREB and ERF subfamilies [5]. *Arabidopsis thaliana* has DREB and the ERF subfamily was additionally split into six segments.

AP2/ERF TFs have been shown to regulate a variety of processes in plants [6]. For example, they regulate plant’s responses to abiotic stress including salt, drought, and cold in addition to normal growth and development [7–9]. AP2 domains were initially described as recurring motifs inside AP2 proteins of *A. thaliana* that mediated a regulatory function during development [3]. Four DNA-binding proteins stimulated by ethylene in tobacco have been implicated in gene transcription, which demonstrated that ERF domains were conserved [9]. ERF proteins were later established to have other roles in several different plants [10]. A total of 145 AP2/ERF are now well recognized in *Arabidopsis* including 64 from the ERF subfamily, 56 from the DREB subfamily, six from RAV, one from the Soloist, and 17 from the AP2 subfamily [5].

Rose (*Rosa chinensis*) is a well-known traditional wooden plant worldwide. Additionally, it is also one of the most important horticultural crops with high economic and ornamental value and has been used for the breeding of cut roses across the globe. With the genome sequencing of diploid *Rosa chinensis* ‘Old Blush’, it can presumably become an excellent model plant for functional genomics of ornamental plants [11]. Many gene families with corresponding growth and development need to be examined. In plants, AP2/ERF proteins are profoundly involved in controlling a plant’s growth and development. With more extensive genomic DNA sequencing being carried out for numerous plants, large number of AP2/ERF proteins have been recognized as yet, including *Apium graveolens* [12], pear (*Pyrus communis*) [13], cauliflower (*Brassica oleracea*) [14], pepper (*Capsicum annuum*) [15], *Salvia miltiorrhiza* [16], *Musa* species [17], Chinese jujube (*Ziziphus jujuba*) [18], buckwheat (*Fagopyum tataricum*) [19] and peach (*Prunus persica*) [20]. However, so far, none of the research studies have been previously conducted on the detection and characterization of AP2/ERF TFs in rose. Therefore, due to the critical contributions of AP2/ERF genes in different biological reactions, a comprehensive study of AP2/ERF in rose is extremely essential.

Initially, our investigation first identified and examined gene sequences, motif structure, promoters, and location of rose AP2/ERF genes on each chromosome. Then, gene duplication and evolutionary mechanisms were subsequently analyzed. Importantly, we also identified a novel DREB A2 subgroup member (i.e., *RcDREB2B*) in rose. RcDREB2B is a nuclear protein and has transactivation in yeast cells. *RcDREB2B* was repressed under mild and severe drought stress in rose root samples. The overexpression of *RcDREB2B* in *Arabidopsis* exhibited enhanced sensitivity to high salt, ABA, and PEG at germination and post-germination stages. Osmotic and ABA-related genes expressions were impaired in *RcDREB2B* transgenic plants. Our findings demonstrated the regulatory roles of rose AP2/ERF TFs in normal homeostasis, which will not only provide a fresh insight into the evolutionary mechanism of this particular TF family in plants but will also contribute towards revealing the molecular mechanisms of development and stress response in rose and other species.

## Results

### Identification and analysis of *RcAP2/ERF*

We used BLASTP and HMMER3.1 in the entire rose genome to discover AP2/ERF members. All potential rose proteins were then subjected to domain analysis to confirm the presence of the AP2 protein domain (PF00847). Eventually, a total of 135 AP2/ERF family members were identified in rose. According to the distribution order of their chromosomal locations, they were named consecutively from *RcAP2/ERF1* to *RcAP2/ERF135.* Their characteristics including gene name, locus ID, physical position, and other properties are shown in Table S1. *RcAP2/ERF* segregated to 7 rose chromosomes and mainly localized in the nucleus, chloroplast, cytoplasm, and mitochondria. The longest sequence with 832 amino acid residues was *RcAP2/ERF52*, whereas the shortest having 125 amino acids was *RcAP2/ERF132*. The predicted isoelectric points ranged from 4.52 (*RcAP2/ERF54*) to 10.28 (*RcAP2/ERF99*). The protein molecular weight varied between 13.94 kDa to 91.01 kDa. Our findings revealed that different biological roles were played by individual coding regions of *RcAP2/ERFs.*

### *RcAP2/ERF* gene structure

**Fig. 1:**
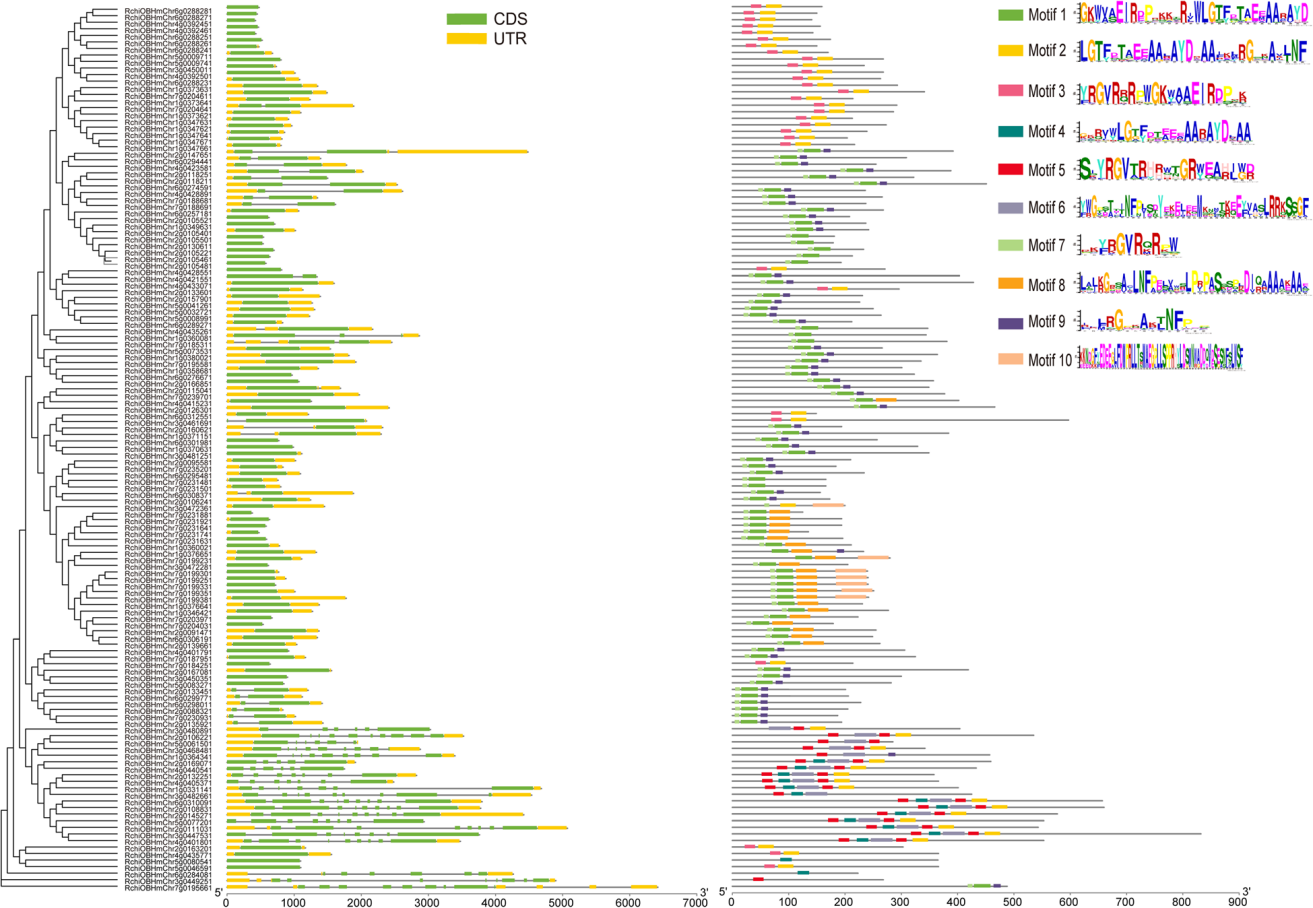
Gene sequence, motif analysis, and phylogenetic association of *Rosa chinensis* AP2/ERF genes. We obtained several alignments of complete amino acid sequences from *Rosa chinensis* by Clustal W and a phylogenetic tree was generated using MEGA 7.0 by the neighbor-joining (NJ) approach with bootstrap repeated 1000 times. Green and yellow boxes are CDS and UTR, respectively. The less complex definition of conserved AP2/ERF family motifs in *Rosa chinensis* genes as explained by MEME analysis. Box colors in each row reflect a conserved motif. Blank lines depict non-conserved motifs

The gene structure of *RcAP2/ERF* is closely related to its function, and together with their phylogenetic analysis, could reflect the phylogenetic relationships among the *RcAP2/ERF*. As shown in Fig. 1, the *RcAP2/ERF* family was divided into seven groups. Analysis of *RcAP2/ERF* structure for exon/intron organizations revealed that each gene had 0 to 9 introns (Fig. 1), the genes clustered into the same branch on the phylogenetic tree were found with similar exon-intron structure. The results provided support validating our analysis regarding gene structure. Further, most *RcAP2/ERF* (94, 69.62%) contained no introns, only 19 (14.07%) contained one intron whereas *RcAP2/ERF30* contained nine introns. It was surprising yet interesting that none of the *RcAP2/ERF* contained three introns. AP2 gene configuration was reasonably conserved among family members, and 51% of AP2/ERF genes had no intron (Fig. 1). Typically, closest family members had a common exon/intron configuration concerning intron number and phase as well as the length of exons.

In an attempt to carry out the in-depth examination of typical RcAP2/ERF protein sequences, we assessed 135 typical arrangements from the MEME website. We predicted that the conserved sequences would amount to 10 in number their sequences were sought (Fig. 1). Besides, we also observed that common patterns with the same orientation and location were most apparent among phylogenetically close relatives. This suggests common roles for AP2 members located in similar subgroups. Motifs 1 and 7, with a net presence of 61.48% (83/135) and 60.74% (82/135) respectively were frequent among RcAP2/ERF. Several preserved motifs have been identified in various subgroups. Phylogenetic investigations show similarity in gene composition and pattern arrangements of subgroup members adding confidence to classification accuracy.

### Phylogenetic and evolutionary analysis of *RcAP2/ERF*

**Fig. 2:**
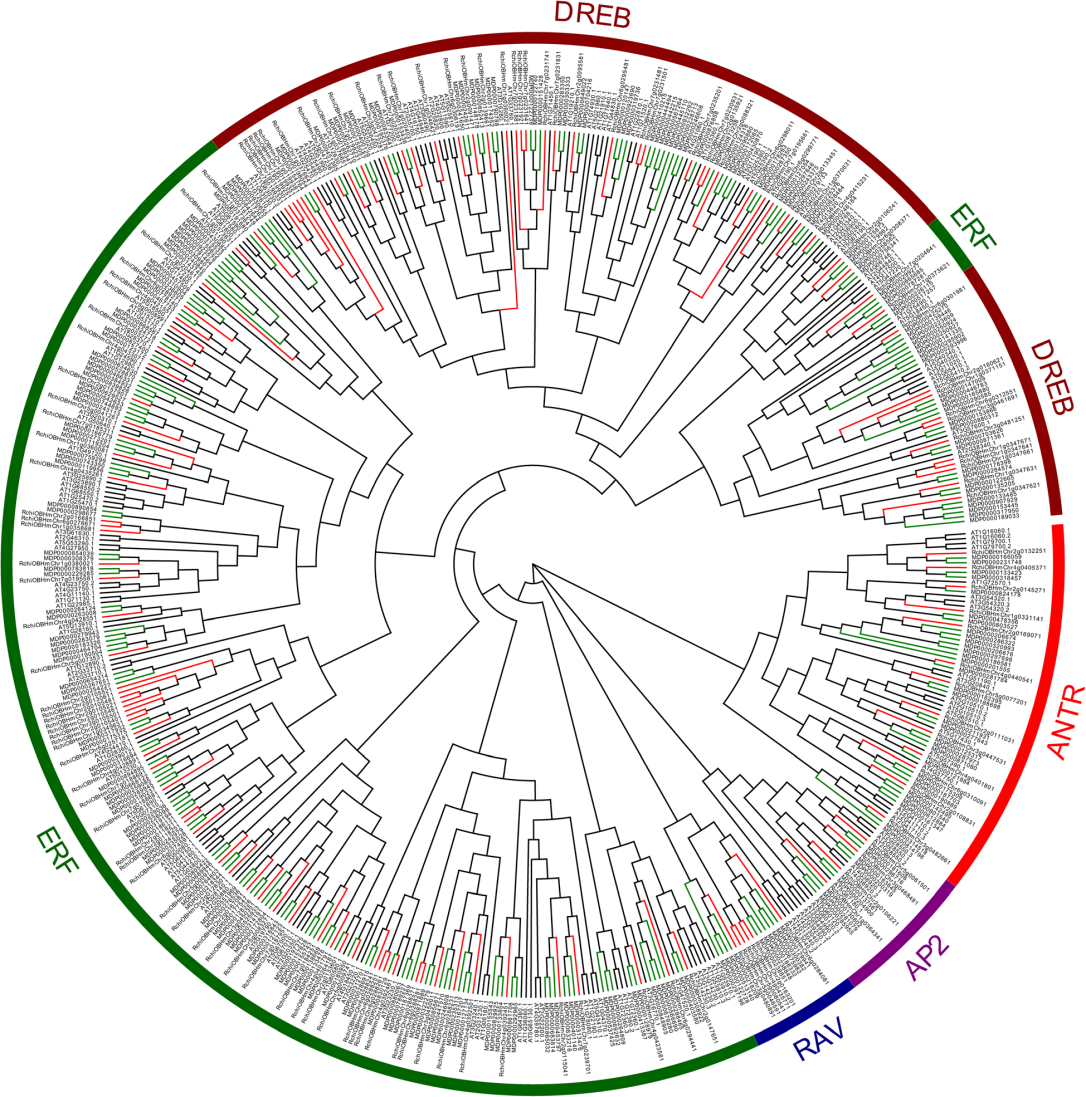
Rose, apple, and Arabidopsis AP2/ERF protein phylogenetic analysis. Highest probability tree built with MEGA 7.0 and Evolview computer programs. Colors and shapes represent AP2/ERF proteins subfamilies from Arabidopsis (black line), apple (green line), and rose (red line). ANTR, AP2, RAV, ERF, and DREB subfamilies are marked with red, purple, blue, green, and red brown color circle lines, respectively

To assess phylogenetic associations of rose AP2/ERF, we aligned AP2 domain sequences of rose, apple (*Malus demostica*), and *Arabidopsis* to construct a parallel phylogenetic tree [21]. One hundred thirty-five RcAP2/ERF proteins of rose, 260 members from apple, and 167 members from *Arabidopsis* were selected for phylogenic analysis (Fig. 2). Using the same criteria for classification as in *Arabidopsis*, the AP2/ERF proteins of rose were classified into DREB, Solosist, AP2, and ERF segments containing 44, 6, 17, and 68 RcAP2/ERF proteins, respectively (Table 1). The *RcAP2/ERF* gene members within groups were mostly the same except ERF-B1, ERF-B6, and ERF-B3. DREB segment is further split into six subgroups, from A1 to A6, containing 7, 11, 1, 15, 7, 3 AP2/ERF proteins in rose and 3, 25, 2, 18, 18, 10 AP2/ERF members in apple, respectively. ERF subfamily being the most abundant type in rose, accounted for 50.37% of all RcAP2/ERF proteins, which was closely similar to apple, with a percentage of 51.54%. *Arabidopsis* has the lowest percentage of ERF subfamily members, which accounted for 46.11%. ERF subfamily was also further segregated to B1–6 subgroups, containing 12, 3, 13, 15, 5, and 20 RcAP2/ERF proteins, respectively. AP2 subfamily contains 17 members of RcAP2/ERF proteins, whereas the Solosist subfamily is the smallest, consisting of only 6 members, indicating that *RcAP2/ERF* genes were distributed in different clades unevenly.

**Table 1:** Summary of AP2/ERF transcription factors of *Rosa chinensis, Malus domestica and Arabidopsis thaliana*

Classification	Group	Number		
		*Rosa chinensis*	*Malus domestica*	*Arabidospis thaliana*
**DREB subfamily**	A1	7	3	7
	A2	11	25	9
	A3	1	2	1
	A4	15	18	16
	A5	7	18	15
	A6	3	10	10
**EFR subfamily**	B1	12	38	18
	B2	3	11	11
	B3	13	25	11
	B4	15	18	12
	B5	5	6	8
	B6	20	36	17
**AP2 subfamily**	AP2-R1	4	9	11
	ANTR1	6	15	8
	ANTR2	7	16	10
**Solosist subfamily**		6	10	3
**Total AP2/ERF family factors**		135	260	167

### Chromosomal location and gene duplication of *RcAP2/ERFs*

**Fig. 3:**
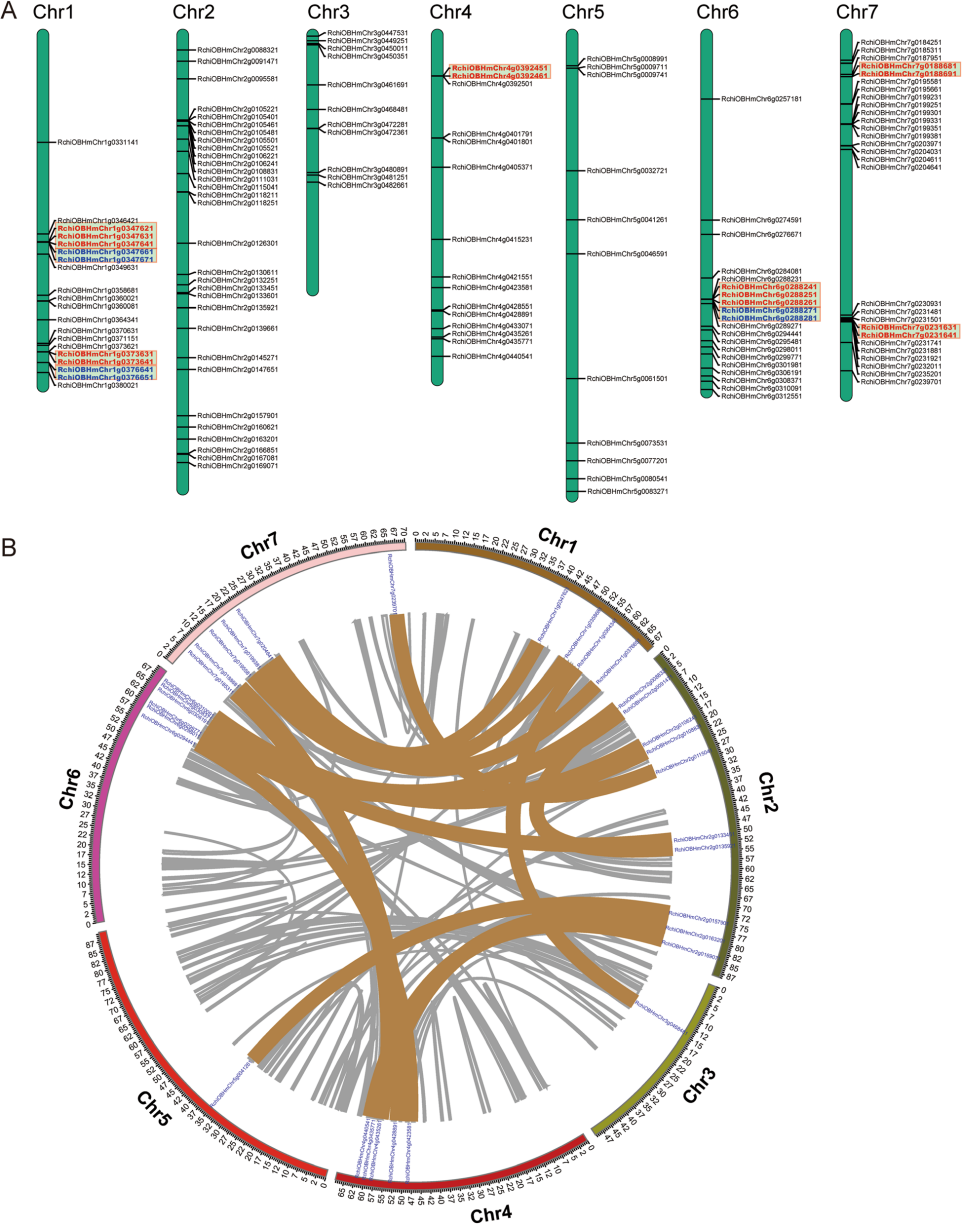
Chromosome mapping and collinearity analysis of *RcAP2/ERF* genes. **a** *RcAP2/ERF* genes distributed on seven rose chromosomes. The brown colored line between the two gene names indicated that they were tandem repeat gene pairs. Gene locations are shown by the scale. The Gene location on each chromosome is represented by a line. **b** Collinearity analysis AP2/ERF gene family in the rose genome. All rose AP2/ERF family duplicated gene pairs are depicted in the rose chromosomes (Chr1–7). The background lines in gray indicate collinear blocks in the whole rose genome, and the collinear relationships of RcAP2/ERF genes are indicated by solid color lines

Mapchart software was used to identify the *RcAP2/ERF* chromosomal location. All *RcAP2/ERFs* had precise positions on the chromosomes. Each rose chromosome contains more than 11 *RcAP2/ERFs* (Fig. 3a). The *RcAP2/ERF* genes on 7 chromosomes are randomly and unevenly distributed. Our maximum predicted the number of *RcAP2/ERF* was 38 genes on chromosome 2 and can be compared to rather fewer, 11 genes located on chromosomes 5 and 3. Although chromosome 3 is the shortest and chromosome 5 is the longest in rose they contain the least *RcAP2/ERF*. Therefore, there is no apparent correlation between chromosome length and *RcAP2/ERF* gene distribution.

Segmental and tandem duplication are evolutionary mechanisms that result in generating groups of genes [22]. Hence, Synteny and MCScanX [23] were used to analyze the duplication and segmental events of *RcAP2/ERF.* As shown in Fig. 3b, 20 genes were confirmed as tandem duplicated genes. Chromosomes 7 and 4 had three groups of two duplicate tandem genes, while chromosomes 6 and 1 had five and four duplicate tandem genes respectively. Segmental duplication events were also detected in each chromosome and accounted for 24.4% of the *RcAP2/ERFs*. Taken in combination, these outcomes indicated that segmental and tandem duplication extensively contributes to the expansion of the RcAP2/ERF family, whereas the former is being more deeply involved in particular.

### Analyzing *cis*-regulatory motifs in promoters of *RcAP2/ERF* genes

**Fig. 4:**
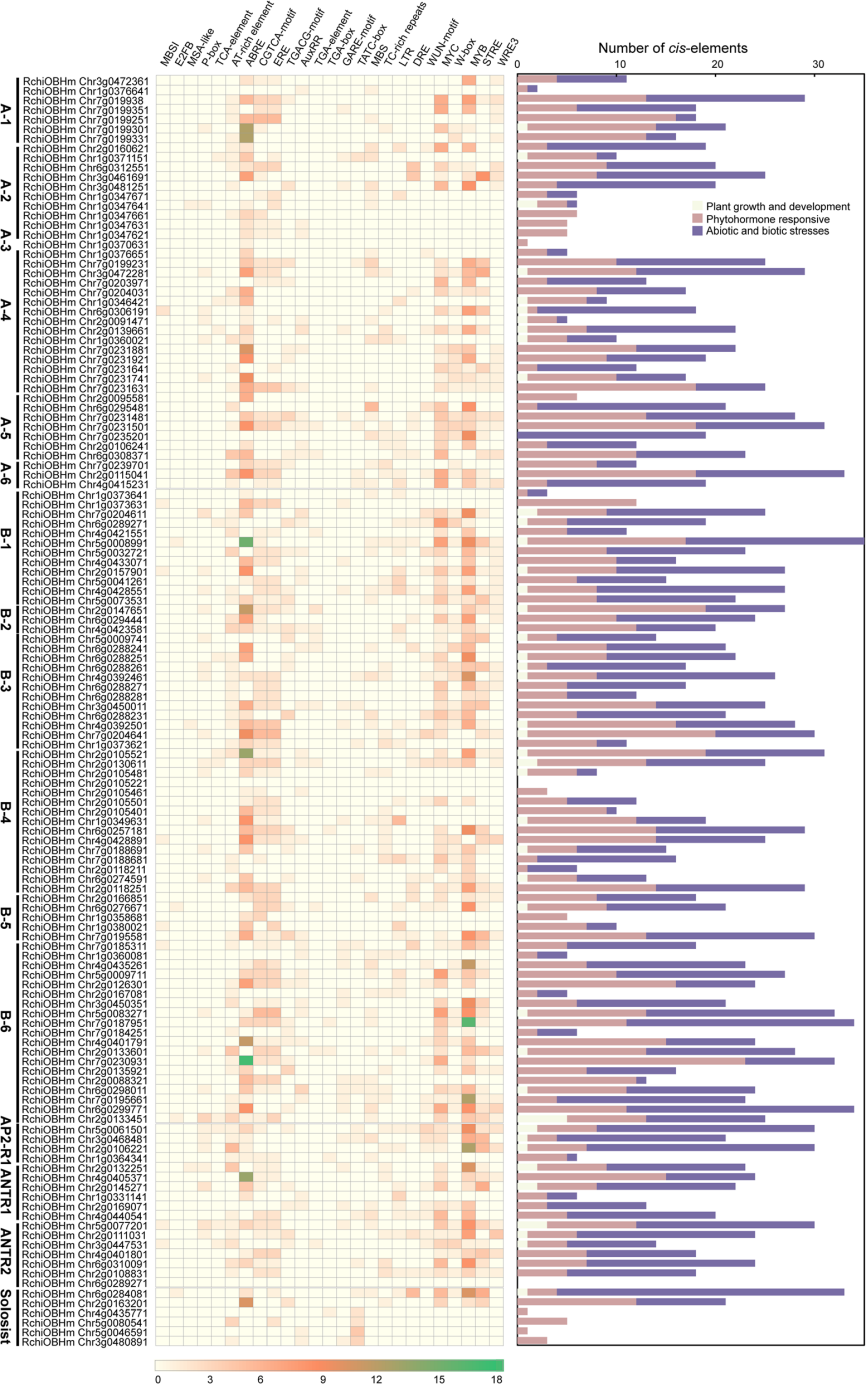
Putative *cis*-elements in *RcAP2/ERF* promoters. The promoter region of *RcAP2/ERF* genes shows *cis*-elements (1500 bp upstream). The assessment was done using PlantCARE, a nucleotide sequence pattern database for plant *cis*-acting regulatory DNA components. Symbols show binding sites. The left panel showed the different AP2/ERF subgroup members. Heatmap represented different *cis*-elements of different *RcAP2/ERFs* promoter regions. MBS I, MYB binding site involved in flavonoid biosynthetic genes regulation; E2F, *cis*-acting element involved in the cell proliferation, differentiation, and endocycle; MSA-like-element, a cell cycle-related element; P-Box, gibberellin-responsive element; TCA-element, *cis*-acting element involved in salicylic acid responsiveness; AT-rich element, the essential elements of replication origins of bacterial replicons; ABRE, *cis*-acting element involved in the abscisic acid responsiveness; CGTCA-motif, *cis*-acting regulatory element involved in the MeJA-responsiveness; TGACG-motif, *cis*-acting element involved in the abscisic acid responsiveness; AuxRR-core, *cis*-acting element involved in the auxin responsiveness; TGA-element, auxin-responsive element; TATC-box, gibberellin-responsive element; MBS, MYB binding site involved in drought-inducibility; TC-rich repeats, *cis*-acting element involved in defense and stress responsiveness; LTR, *cis*-acting element involved in low-temperature responsiveness; DRE, *cis*-acting element involved in dehydration, high salinity, or low temperature; WUN-motif, wound-responsive element; MYC, *cis*-acting element involved in the dehydration and abscisic acid responsiveness; W box, WRKY binding site; MYB, *cis*-acting element involved in the dehydration and abscisic acid responsiveness; STRE-element, stress-responsive element; WRE3-element, *cis*-acting regulatory element involved in high temperature. Three different types of *cis*-acting elements (plant growth and development, phytohormone responses, and abiotic and biotic stresses) are represented by different colors, as shown on the right

To establish the gene expression control of *RcAP2/ERF*, a bioinformatics review was performed to detect apparent *cis*-regulatory regions in the *RcAP2/ERF* gene promoter region. This promoter zone was identified by the ~ 1500 bp upstream area of the initial transcript sites. Figure 4 illustrates that different promoter *cis*-elements were grouped into three groups, including plant growth and development (MBSI, E2FB, MSA-like, P-box, TCA-element, AT-rich element,), phytohormone responsive (ABRE, CGTCA-motif, ERE, TGACG-motif, AuxRR, GARE-motif, TATC-box), and abiotic and biotic stresses (MBS, TC-rich repeats, LTR, DRE, WUN-motif, MYC, W-box, MYB, STRE, WRE3). These *cis*-regulatory elements were found to be randomly dispersed among various promoters of *RcAP2/ERF* genes. ERF subfamily B1 group member *RchiOBHmChr5g0008991* has the largest number of *cis*-elements, accounting for 43. MYB and MYC elements were present in 79.4% of *RcAP2/ERF* gene promoters, whereas 77.9% were found with ABRE. Moreover, 66.2, 50.7, 45.6, and 44.9% of RcAP2/ERF gene promoters were presenting MeJA, gibberellin, W box and MBS, respectively, which indicating important contributions to gene expression of the *RcAP2/ERF* gene. However, *RcAP2/ERF* gene promoters had a lower abundance of some motifs such as LTR (33.8%), DREB (32.4%), and salicylic acid responsiveness (31.6%), suggesting that these regions controlled the expression of particular genes only under appropriate circumstances. These analyses of *cis*-regulatory elements implied that *RcAP2/ERF* genes may not only be involved in hormone signaling but also participated in reacting to external stresses.

### Isolation, structure, and promoter analysis of *RcDREB2B*

As DREB A2 subgroup members are considerably involved in abiotic stress tolerance in different plant species, we select *RcDREB2B*, which belongs to DREB A2 subgroup member, for further analysis. The primers of *RcDREB2B* were designed for PCR amplification, and full-length cDNA sequences were cloned. The sequences of *RcDREB2B* cDNA and deduced amino acid were submitted to the National Center for Biotechnology Information (NCBI) GenBank (MH152409). It was revealed by sequence analysis that an open reading frame (ORF) of 585 bp was contained in the full-length cDNA and encoded a putative protein of 194 amino acids with a pI of 9.03 and a predicted molecular weight of 21.2 kDa.

It is evident from multiple sequence alignment between reported DREB A2 subgroup members (AtDREB2B, TaDREB1, ZmDREB2A, and HvDRF1) and RcDREB2B that all proteins demonstrate the characteristics features of DREB proteins, specifically a conserved DNA-binding domain (AP2-domain) comprising 64 amino acids, and conserved YRG, WLG, and RAYD motifs. Glutamic acid (E19) was conserved at the 19th position in the AP2 domain in RcDREB2B, HvDRF1 and TaDREB1 (Additional file 3: Figure S1). Moreover, the AP2 domain manifested a greater degree of amino acid identity than the N-termini and C-termini of the proteins. There was also a typical nuclear localization signal (NLS) in the N-terminal region (Additional file 3: Figure S1). The sequence analysis revealed that RcDREB2B possessed a high degree of sequence homology to AtDREB2B (30% identity) in *Arabidopsis thaliana*, TaDREB1 (36.49% identity) in *Triticum aestivum*, HvDFR1 (26.61% identity) in *Hordeum vulgare*, and ZmDREB2A (30.09% identity) in *Zea mays*.

**Fig. 5:**
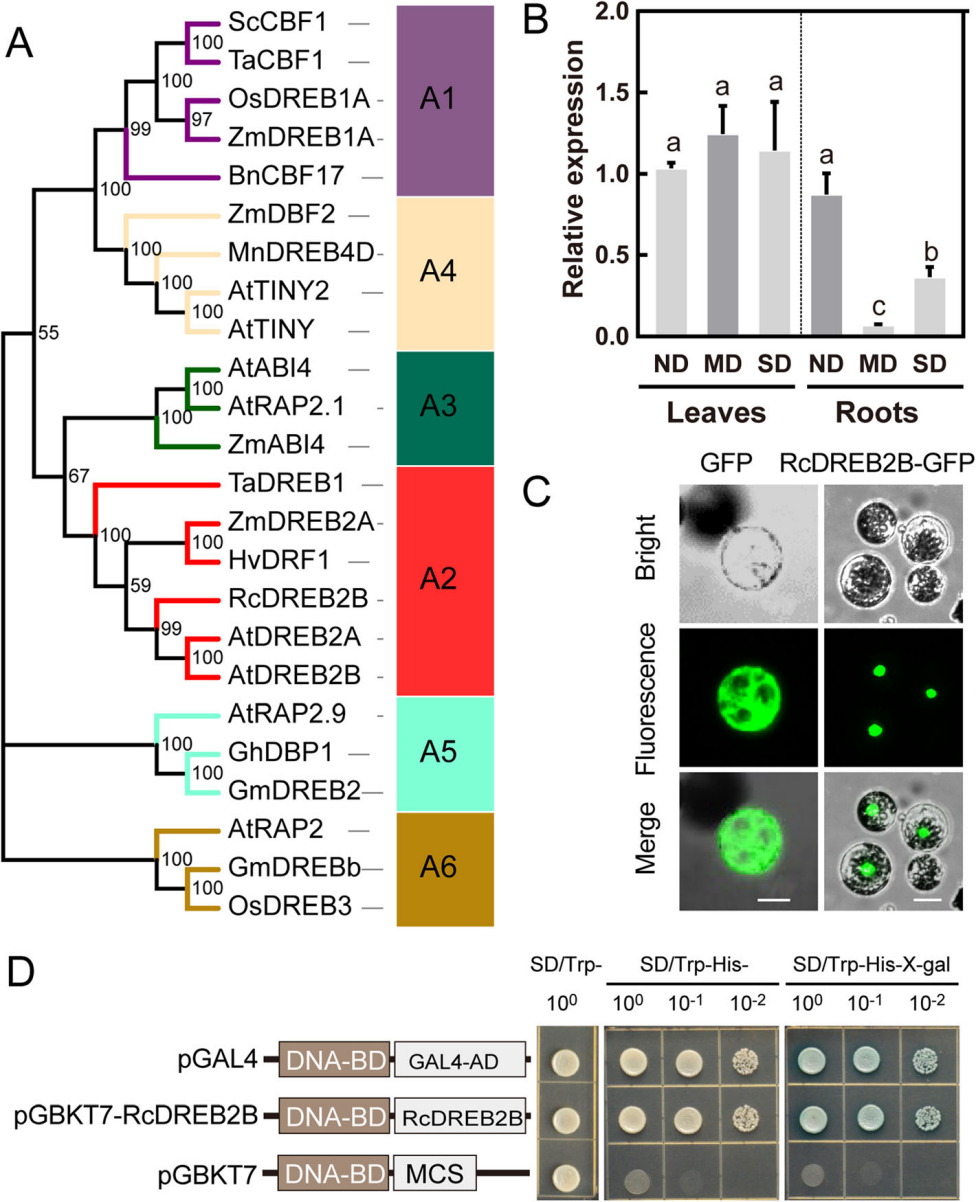
Characteristics of RcDREB2B. **a** Phylogenetic tree of RcDREB2B and other plant DREB proteins. The phylogenetic tree was constructed using Evolview and MEGA 7.0 with bootstrap values of 1000 replicates. The accession numbers of the protein sequences that have been retrieved from NCBI and species designations are listed below: *Arabidopsis thaliana*: AtDREB2A (AB007790), AtDREB2B (NM_111939.2), AtABI4(A0MES8), AtTINY2 (AY940160.1), AtTINY(Q39127), AtRAP2.1 (Q8LC30), AtRAP2.9 (NM_179009.1), AtRAP2 (AAP04063.1); *Oryza sativa*: OsDREB1A (AF300970), OsDREB3 (NP_001048142); *Glycine max*: GmDREB2 (ABB36645), GmDREBb (AAQ57226); *Gossypium hirsutum*: GhDBP1(AAO43165.1); *Zea mays*: ZmDREB1A (AF450481), ZmDREB2A (NP_001105876), ZmABI4 (AY125490), ZmDBF2 (AF493799); *Hordeum vulgare*: HvDRF1 (AY223807); *Triticum aestivum*: TaDREB1 (DQ195068), TaCBF1 (AF376136); *Secale cereal*: ScCBF1 (AF370730); *Brassica napus*: BnCBF17 (AF499034); *Morus notabilis*: MnDREB4D (AHJ25980.1). Different color lines indicate different subgroups of the DREB subfamily. **b** Transcript levels of *RcDREB2B*. The expression levels of *RcDREB2B* in leaves and roots during no drought (ND), mild drought (MD), and severe drought (SD) treatments. The mean fold changes of *RcDREB2B* in MD and SD were compared with ND. We present results as means ± SD. *n* = 3. Different letters denote significant differences at *P* < 0.05 with one-way ANOVA analysis. **c** Subcellular localization of RcDREB2B in Arabidopsis protoplast cells. RcDREB2B-GFP fusion protein or GFP alone is expressed under the control of pCAMBIA 1300 in *Arabidopsis* protoplast cells. The photographs were taken in the green channel, bright channel, and merge channel. Bars = 20 μm. **d** Transcriptional activity analysis of RcDREB2B. The full-length ORF of RcDREB2B was fused to the GAL4 DNA-binding domain in the vector pGBKT7 to generate pGBKT7-RcDREB2B, and the construct was converted into yeast strain Y2HGold. Yeast dilutions were grown on SD medium lacking Trp, and the same medium containing with or without X-gal but lacking with Trp, His, Ade. pGBKT7 and pGAL4 plasmids were respectively used as negative and positive control

Based on the amino acid sequences of RcDREB2B and other plants’ DREB proteins, a phylogenetic tree was constructed. Phylogenetic analyses revealed that DREB proteins can be categorized into six sub-groups (A1–A6), among which RcDREB2B along with AtDREB2A, AtDREB2B, TaDREB1, ZmDREB2A, and HvDRF1 belong to the A2 sub-group (Fig. 5a). Moreover, RcDREB2B exhibited high similarity to AtDREB2A and AtDREB2B. Therefore, this gene was named RcDREB2B instead. Collectively, the data implies that RcDREB2B is an A2 sub-group member of the DREB subfamily.

### Expression, nuclear localization, and transcriptional activity of RcDREB2B

We analyzed *RcDREB2B* expression in roots and leaves of the rose seedlings under normal drought stress (ND), medium drought stress (MD), and severe drought stress (SD) by real-time quantitative PCR (RT-qPCR). *RcDREB2B* was constitutively expressed in leaves tissues under the treatments we tested. In the root tissue, expression levels of *RcDREB2B* were significantly down-regulated under MD and SD treatment, in comparison to the ND controls (Fig. 5b). These findings indicated that *RcDREB2B* might be involved in mediating drought stress signaling transduction.

Investigation of the subcellular localization of RcDREB2B was conducted by constructing GFP and RcDREB2B-GFP and transformed them with *Arabidopsis* protoplast cells. Figure 5c shows that GFP fluorescence is equally divided in the nucleus and the cytoplasm with the control plasmid GFP, whereas the RcDREB2B-GFP fusion protein, was only located in the nucleus (Fig. 5c). A yeast assay system was used to estimate whether RcDREB2B was capable of activating transcription. Yeast strain Y2HGold was transfected individually with pGBKT7-RcDREB2B, pGAL4 (positive control), and pGBKT7 (negative control) plasmids. All transformed yeasts were able to grow in SD/Trp- media. The yeast transfected with pGBKT7-RcDREB2B and pGAL4 was able to grow on the SD/Trp-His-Ade-, however the negative control was not able to grow. Besides, blue color was observed upon incubation of the yeast extract of yeast transfected with pGBKT7-RcDREB2B and pGAL4 with X-gal (Fig. 5d). The outcome implies that RcDREB2B is a nuclear-localized transcriptional activator.

### Overexpression of *RcDREB2B* reduced the high salinity tolerance in transgenic *Arabidopsis*

**Fig. 6:**
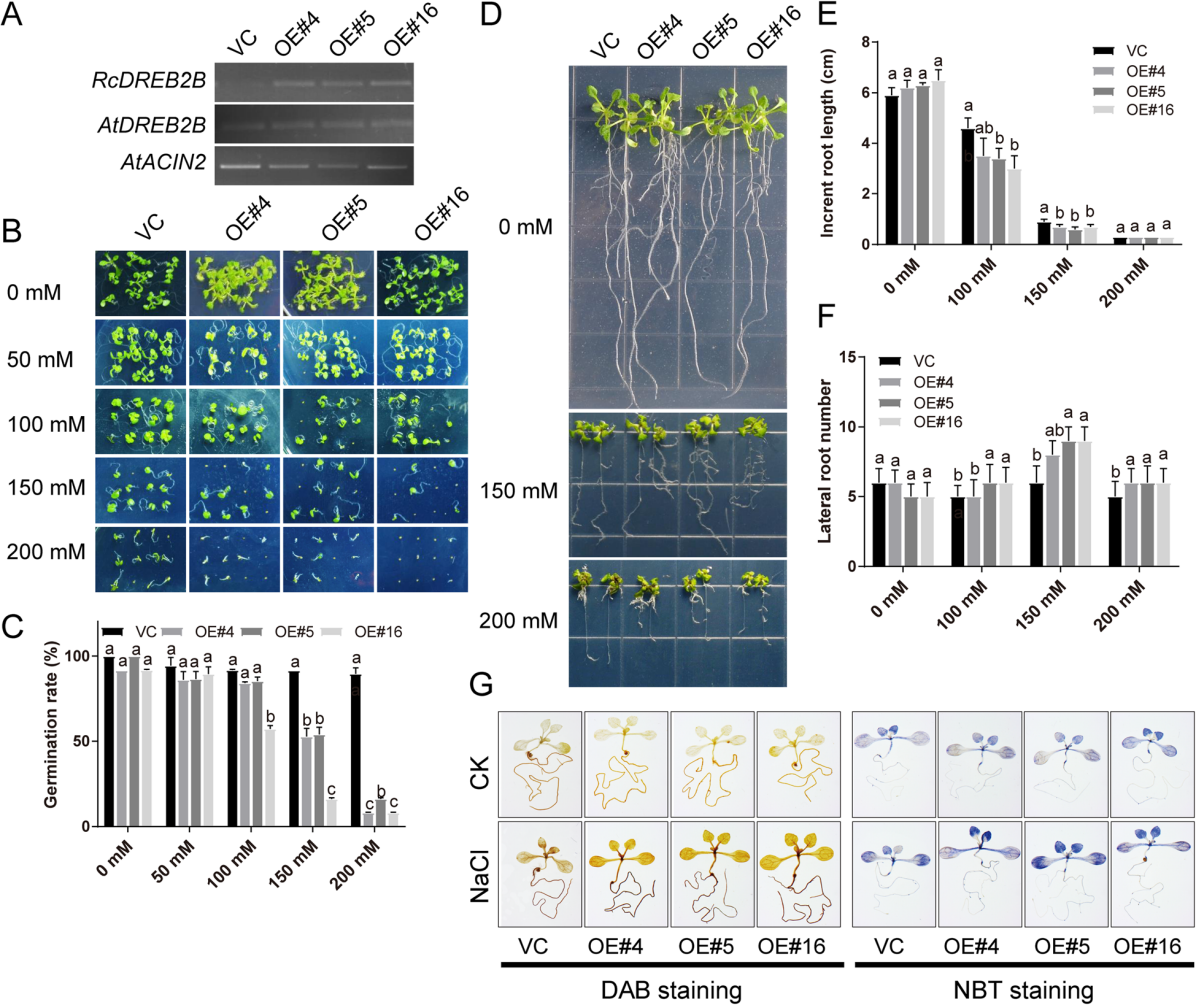
Seed germination and post-germination assays of VC and *RcDREB2B* overexpressed lines in response to NaCl. **a** RT-PCR analysis of *RcDREB2B* and *AtDREB2B* in the overexpressed lines. The specific primers of *RcDREB2B* were used to test transgene levels. *AtACTIN2* was used as an internal control. Uncropped gel images are provided in Additional file 7: Figure S5. **b** Seed germination performance of *RcDREB2B* overexpressed lines (OE#4, 5, 16) and VC on MS supplement with various NaCl concentrations (0, 50, 100, 150, and 200 mM). **c** Germination rates of seeds indicated in (**b**). Seed germination was monitored with radicle emergence and cotyledon greening after 9 d of post-stratification. Twenty seeds from VC and *RcDREB2B* transgenic lines were used with three biological replicates and means of + SE. Different letters stand for *P* < 0.5, using one-way ANOVA analysis. **d** Root growth performance of *RcDREB2B* overexpressed lines (OE#4, 5, 16) and VC in response to NaCl. 6 d old seedlings of VC and *RcDREB2B* transgenic plants were placed on MS plates supplemented without and with 150 or 200 mM NaCl for 10 days, respectively. Comparisons of increment in root length (**e**) and lateral root numbers (**f**) of VC and *RcDREB2B* overexpressed lines. Data indicate means + SE of three biological replicates. Different letters indicate *P* < 0.5, using one-way ANOVA analysis. **g** Histochemical staining assay detecting O_2_^−^ and H_2_O_2_ accumulation with nitro blue tetrazolium (NBT) and 3,3-diaminobenzidine (DAB) in VC and *RcDREB2B* transgenic seedlings under normal growth or salt stress. We present results as means ± SD. *n* = 3

Driven by a constitutive super promoter [24], *RcDREB2B* was overexpressed in *Arabidopsis* for further functional characterization. The T3 generation of three independent *RcDREB2B*-overexpression lines (OE#4, 5, and 16) with different expression levels was subsequently chosen for analysis (Fig. 6a). No apparent morphological changes in terms of flower diameter, petal length, petal width, and single petal area were observed between VC and *RcDREB2B*-OE plants (Additional file 4: Figure S2). MS media with 0, 50, 100, 150, and 200 mM NaCl were used to sow seeds of the *RcDREB2B*-OE and VC (Fig. 6b). No significant morphological differences were observed in the rate of seed germination between *RcDREB2B*-OE and control lines showed when grown on 0, 50, and 100 mM NaCl plates. However, when supplemented with 150 and 200 mM NaCl, the germination rate of all plants reduced, accordingly. Upon supplementation with 150 mM NaCl, the germination rates of OE# 4, 5, and 16 were 52.8, 54.1, and 16.2%, respectively, significantly lower than VC controls (91.7%) (Fig. 6b, c).

To further analyze the root phenotypes of *RcDREB2B*-OE plants under salt stress, we placed 6-day-old seedlings in the MS media plates containing 0, 100, 150, and 200 mM NaCl, and treated for 10 days (Fig. 6d). The increment root length between *RcDREB2B*-OE and VC exhibited no significant variation when grown on 0 and 200 mM NaCl plates. But, at NaCl concentration of 100, or 150 mM, the VC displayed a significantly longer increment of root length than *RcDREB2B*-OE lines (Fig. 6e). For instance, when the growth was carried out on MS plates containing 150 mM NaCl, the increment in root length of the VC plants was 0.88 cm, while the increment in root lengths of the OE# 4, 5, and 16 was 0.70, 0.61, and 0.70 cm, respectively (Fig. 6e). Without NaCl treatment, there were no significant differences between *RcDREB2B*-OE lines and VC controls in terms of lateral root number. However, the lateral root number the same in *RcDREB2B*-OE lines was significantly more than VC in plants when exposed to the 150 and 200 mM NaCl condition (Fig. 6d, f).

Next, two distinct histochemical staining assays were carried out on *Arabidopsis* seedlings to detect O_2_^−^ and H_2_O_2_ using DAB and NBT, respectively. Ten-day-old seedlings of *RcDREB2B* transgenic and VC plants were exposed to 200 mM NaCl or water (CK) solutions for 1 h. No significant differences in histochemical staining of seedlings leaves and roots were observed in both *RcDREB2B*-OE and VC plants under CK conditions (Fig. 6g). Compared with VC, *RcDREB2B*-OE lines showed significantly stronger blue and brown color under 200 mM NaCl. Quantities analyses of H_2_O_2_ and O_2_^−^ indicated *RcDREB2B*-OE exhibited significantly higher content than VC controls (Additional file 5: Figure S3). The results demonstrate that *RcDREB2B*-OE accumulated higher H_2_O_2_ and O_2_^−^ levels compared with VC plants under salt stress, thus implying that *RcDREB2B* plays a negative role in controlling reactive oxygen species. These results demonstrate that overexpressing *RcDREB2B* in *Arabidopsis* decreased salt tolerance during the seedling stage as well as germination.

### Overexpression of *RcDREB2B* leads to ABA sensitivity in *Arabidopsis*

**Fig. 7:**
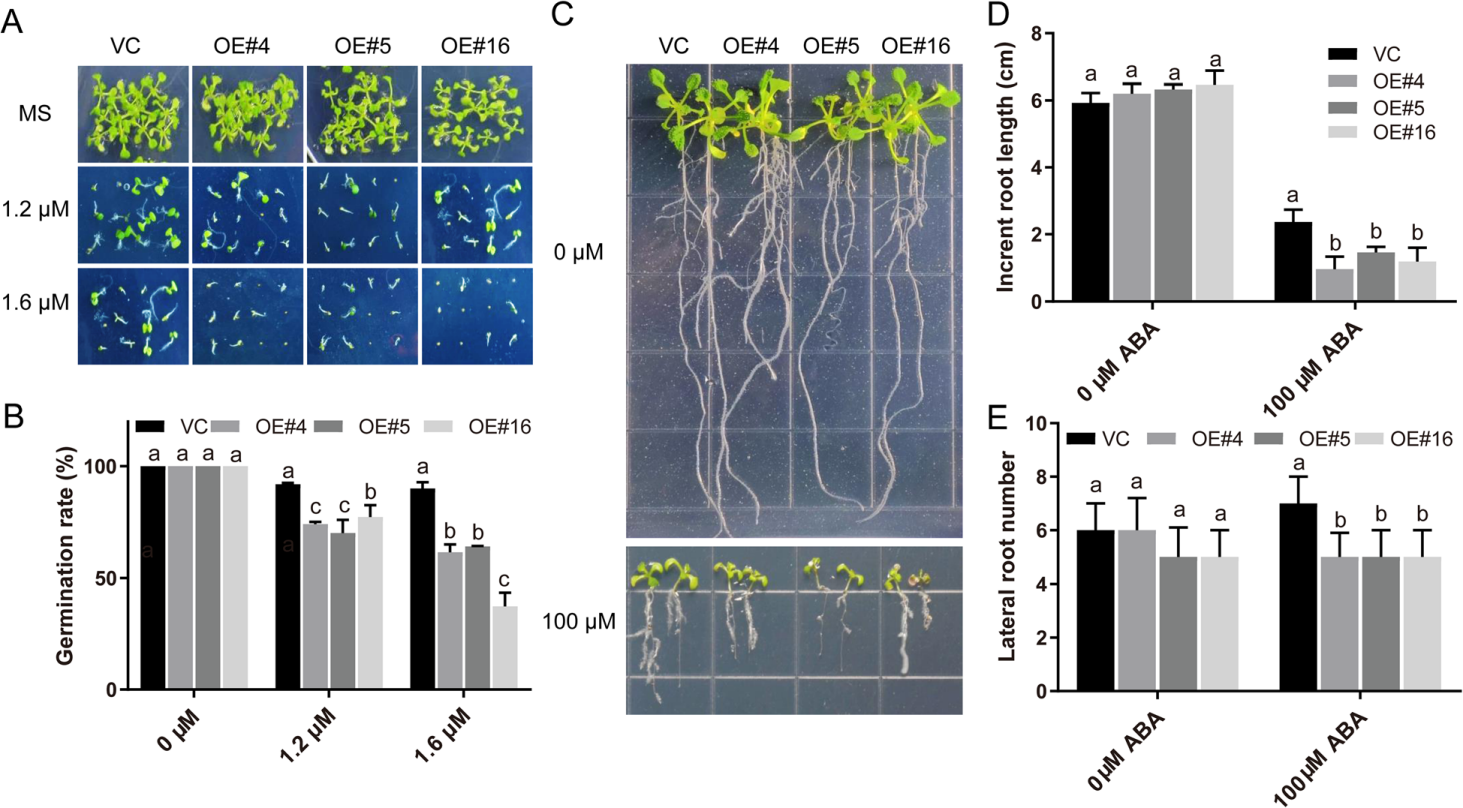
Germination and root growth analysis of VC and *RcDREB2B* overexpressed lines in response to ABA. **a** Seed germination performance of VC and *RcDREB2B* overexpressed lines on MS with different ABA concentrations (0, 1.2 μM, 1.6 μM) after 9 days. **b** Statistical analyses of germination rates are indicated in (**a**). Seed germination rates were measured after 9 d growth. **c** Root growth of VC and transgenic seedlings on MS agar plates containing 0 μM or 100 μM ABA. Representative photos were taken following 10 days of the transfer. Seedlings were 6-days-old at the time of transfer. Increment in root length (**d**), and lateral root number (**e**) of VC and *RcDREB2B* transgenic plants in response to ABA. Different letters indicate significant differences in three independent experiments at *P* < 0.05 using one-way ANOVA analysis

To elucidate the involvement of *RcDREB2B* in ABA signaling, we conducted a phenotypic analysis of *RcDREB2B*-OE plants at the germination and root development stages in response to ABA (Fig. 7). Seeds were sown on MS medium supplemented with different concentrations of ABA (0, 1.2, and 1.6 μM) for 9 days (Fig. 7a). In the absence of ABA, the germination rates of *RcDREB2B*-OE and VC plants were 100%. However, with 1.2 μM ABA, the germination rates decreased to 70.05–77.22% for *RcDREB2B*-OE lines, and 91.98% for VC. Similar inhibition was observed in these plants grown on a 1.6 μM ABA plate, the germination rates of OE#4, OE#5, and OE#16 were 37.4, 64.1, and 61.5%, respectively, whereas VC germination rate was 90.0% (Fig. 7b). We also tested the root phenotype of *RcDREB2B*-OE and VC exposed to 0 and 100 μM ABA (Fig. 7c). However, no significant differences were observed in the root phenotype of *RcDREB2B*-OE and VC seedlings in the absence of ABA. As shown in Fig. 7d, the *RcDREB2B*-OE plants exhibited a 0.97–1.20 cm increment in root length under 100 μM ABA in comparison to 2.4 cm in VC controls. *RcDREB2B* transgenic plants had a significantly lesser lateral root number as compared to VC in the presence of 100 μM ABA (Fig. 7e). Such results suggest that *RcDREB2B* causes enhancement in ABA sensitivity of plants during germination and thereafter.

### Overexpression of *RcDREB2B* exhibit sensitivity to inhibition of germination by osmotic stress in transgenic *Arabidopsis*

Since the sensitivity of plants towards ABA at the seed germination stage of *RcDREB2B*-OE has been increased, we predicted that overexpression of *RcDREB2B* may also influence plant tolerance to stress. In an attempt to investigate this, the seed germination of *RcDREB2B*-OE and VC controls under 0, 4, 8, 12, and 16% PEG treatment was analyzed (Additional file 6: Figure S4). *RcDREB2B*-OE and VC lines when grown on 0, 4, and 8% PEG plates showed no significant differences. The germination rate of *RcDREB2B*-OE and VC controls were significantly inhibited by 12 and 16% PEG, but the inhibition in the VC was less severe. When supplemented with 12% PEG, VC germination rates declined to ~ 92.28%, whereas *RcDREB2B*-OE lines displayed 75.6–78.6% germination (Additional file 6: Figure S4). Furthermore, *RcDREB2B*-OE lines displayed a 67.5–71.7% germination ratio in MS medium supplement with 16% PEG whereas VC germination was reduced to 93.0% (Additional file 6: Figure S4). It is evident from these results that *RcDREB2B*-OE seeds display a sensitivity to inhibition of germination upon stress due to osmosis.

### Overexpression of *RcDREB2B* in *Arabidopsis* altered the expression of osmotic and ABA-responsive genes

**Fig. 8:**
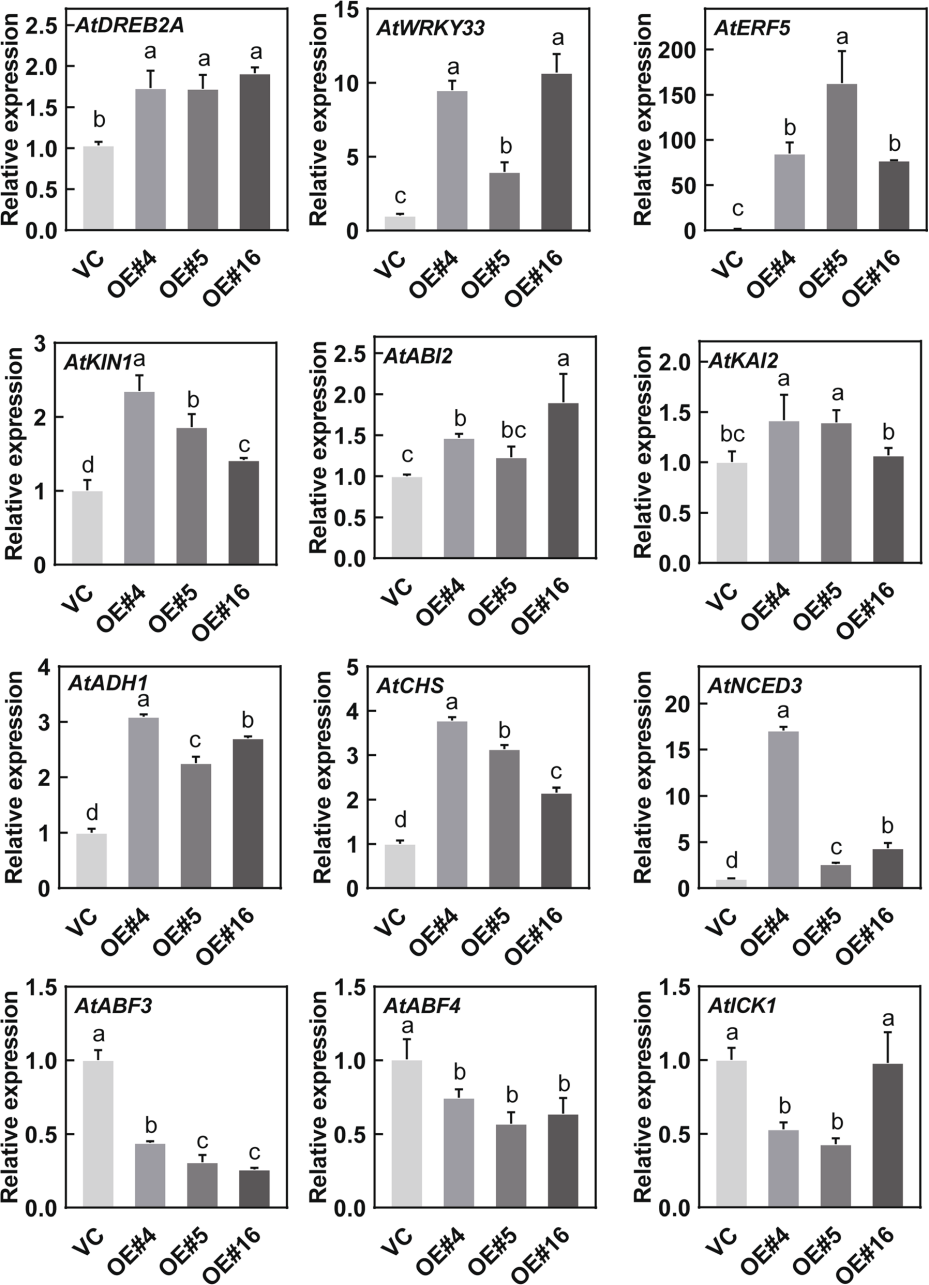
Expression analysis of osmotic and ABA-related genes in VC and *RcDREB2B* overexpressed lines. Analysis of the osmotic stress-responsive genes and ABA-related genes in VC and *RcDREB2B* transgenic lines was performed using RT-qPCR. Total RNA extraction was carried out from 3-week-old seedlings grown under normal growth conditions. Three separate replicates were carried out for RT-qPCR analysis. The relative expression of individual genes of VC was set to 1.0, and *AtACTIN2* was used as an internal control gene. An additional file (Additional file 2: Table S2) lists the primers used. Different letters indicate significant differences in three independent experiments at *P* < 0.05 using one-way ANOVA analysis

To further explore the molecular mechanisms through which *RcDREB2B* controls the sensitivity of germination, RT-qPCR analysis was carried out to examine the expression pattern of four osmotic stress-responsive genes (*AtDREB2A* [25], *AtWRKY33* [26], and *AtERF5* [27], *AtKIN1* [28]), seven ABA-responsive genes (*AtABI2* [29], *AtKAI2* [30], *AtADH1* [31], *AtCHS* [32], *AtABF3* [33], *AtABF4* [34], and *AtICK1* [35]), one ABA biosynthesis gene (*AtNCED3* [36]) in *RcDREB2B*-OE and VC plants under normal growth condition. Relative expression of *AtDREB2A*, *AtWRKY33*, *AtERF5*, *AtKINI*, *AtABI2*, *AtKAI2*, *AtADH1*, and *AtNCED3* was significantly increased in the *RcDREB2B*-OE plants under normal conditions compared with VC controls. Besides, the chalcone synthase gene *CHS* [32], which is classified as the stress-responsive biosynthetic genes, was up-regulated. But, two abscisic acid-responsive element-binding factors (*AtABF3*, *AtABF4*) exhibited a significant reduction under normal conditions (Fig. 8). The enhanced or attenuated expression of these genes in *RcDREB2B* might contribute to the ABA-induced sensitivity to inhibition of germination as well as salinity and drought stress during the period of seed germination and early seedling development.

## Discussion

Studies of TFs have been evolved to become an integral element in functional genomics research. TFs, with the involvement of various genes, mediate stress responses by inducing activating or inhibitory signaling pathways. Plants, thereby, make use of these mechanisms to adapt to different environmental conditions [37]. Among plant TFs, the AP2/ERF family is one of the largest concerning the size. AP2 is known to be involved in plant reproduction and homeostasis particularly in plants’ responses to biological or non-biological stresses [8]. Genome-wide scan of AP2 has been systematically carried out in *Arabidopsis* [5], *Fagopyum tataricum* [19], *Setaria italic* [38], *Vitis vinifera* [39], peach (*Prunus persica*) [40], castor bean (*Ricinus communis*) [41], and *Salix arbutifolia* [42]. Despite this, the information on the AP2/ERF genes of rose is still scant. Herein, we identified and investigated the information regarding AP2/ERFs in rose. Our study revealed, that a total of 135 AP2/ERFs were identified, including 44 DREB, 68 ERF, 17 AP2, and 6 Solosist subfamily members, possessing identical structural features with other plants. However, in different species, the numbers of AP2/ERFs varied largely. *Arabidopsis* contains 167 AP2/ERF genes, *V. vinifera* contains 132 AP2/ERF genes, *F. tataricum* contains 135 AP2/ERF genes, and peach contains 130 members. The genome of these plants varies greatly in terms of size: rose (600 Mb), *V. vinifera* (475 Mb), peach (265 Mb), and *F. tataricum* (489 Mb), which shows stable AP2/ERF superfamily member numbers with no direct relation to genome size.

Analysis of domain conservation and gene sequences revealed pattern conservation in *RcAP2/ERF* genes from each group. The preserved patterns and intron-exon distribution of *RcAP2/ERFs* were analyzed to obtain a better understanding of the structural features of rose AP2/ERFs. While introns were absent in 68.38% of RcAP2/ERF, AP2 subgroup genes had between 1 and 9 introns. The conserved motif analysis of *RcAP2/ERFs* supported the indicated phylogenetic relationship and classification of rose AP2/ERFs. Rose AP2 gene composition was found to resemble that of *F. tataricum* [19] and *Hordeum vulgare* [43]. AP2 structural subgroup gene differences suggested the presence of numerous evolutionary gene changes. Conserved TF motifs played an important role in the work of particular groups, as recognized in DNA binding, transcriptional behavior, and protein interaction [37]. The examination of motifs demonstrated that the majority of rose AP2/DREB subfamily genes expressed motifs 1, 7, 8, and 10 (Fig. 1). Motifs 1, 2, 3, and 7 have been found in different ERF system groups, suggesting that they have valuable functions associated with them. Results have shown that, while several variations of the AP2/ERF family genes have been significantly conserved, emerging evolutionary motifs that carry out novel activities in some plants need more research. Two patterns were found relatable to the AP2 domain, that is, motifs 1 and 2. Motif 1 covered the largest area of the AP2 domain (PF00847), even Gx4E, WLG, and AYD components. While motif 2 retained the AYD group. All *RcAP2/ERFs* bore a minimum of one of the two patterns that exhibit high conservation of the AP2 domain in genes of *RcAP2/ERF*. Besides the AP2 domain-related motifs, there were another eight motifs outside the AP2 domain in the group-specific distributions. Motif 8 was expressed by members of group A4 of the DREB subfamily, distinguished by four chains of preserved amino acid residues: LNFP, D [IV] QAA / DIR [RA], and LPRP (Fig. 1). Preserved amino acids have been identified as critical sites in *Arabidopsis* for serine / threonine-proteins kinase-12 that binds CBL [44], dehydration-responsive item binding protein-1C and protein-G [45], ARF19 [45], disease resistance [46], and ERF037 [44], respectively. Nevertheless, the roles of other motifs are still unclear, and an extensive amount of investigation is required to understand their biological functions.

Previous studies have reported gene duplication as a major source of evolution of the gene family, including individual gene duplications, segmental duplication, tandem duplication, chromosomal fragments, transposition events, and entire genomes duplication [47]. Furthermore, segmental duplication and translocation have enabled plants to rapidly adapt to any change in the environments [48]. To acquire a better understanding of the expansion mechanism of AP2 genes, we carried out an analysis of the gene duplication events in rose. We identified at least nine pairs of *RcAP2/ERF*, including three tandem duplications and two segmental duplications. The *RcAP2/ERF* genes are randomly and unevenly distributed on 7 chromosomes. Segmental duplication events were also detected in each chromosome and accounted for 20% of the *RcAP2/ERF*. Our findings have shown that gene replication influences *RcAP2/ERF* chromosomal position and gene family extension depends on sequence duplication, either of the tandem or segmental kind.

Gene expression specificity depends on *cis*-regulatory elements and their associations with TFs [49]. Fifteen *cis*-acting regulatory elements were found in *RcAP2/ERF* roses, including ABRE, LTR, MYB, MYC, and DRE, and were designated to 1.5 kb upstream of the *RcAP2/ERF* initiation codon. These *cis*-elements are active in the control of genes under abiotic or biotic stresses. The ABRE element class binds to ABA-dependent TFs which are conservatively strong. Their promoters also contain stress-resistant genes such as those related to dehydration, drought, salt, and low temperature. In response to non-biological stress, they regulate the expression of associated genes [50]. *Cis*-acting ABRE regulatory elements are present in most upstream regulatory sequences of *RcAP2/ERF*, suggesting that these genes play an indispensable part in reacting to environmental stimuli. The LTR element which mediates the response to low temperatures is also *cis*-acting [51]. *RchiOBHmchr1g0331141* and *RchiOBHmchr1g0349631* possess three continuous repetitive LTRs, suggesting that these two genes can operate under low-temperature responses. MYB *cis*-element is the DNA binding site for MYB transcription factors and regulates defense, abiotic and biotic stress reactions, differentiation, development, metabolism, etc. [52]. Within the *RchiOBHmchr3g0449251* promoter regions, many repeated MYB *cis*-elements have been identified, indicating that *RchiOBHmchr3g0449251* is a putative direct target of MYB transcription factors. Most *RcAP2/ERF* carries one or more W-BOX *cis*-elements, where DNA binding occurs for WRKY transcription factors. They are also involved in the genetic control of plant reproduction and homeostasis, leaf aging, cell signaling, and many abiotic and biotic stress reactions [53]. When the *cis*-regulatory elements were examined they showed, that the promoter sequences of the *RcAP2/ERF* genes were not uniformly distributed. MYB and MYC *cis*-regulatory zones were most abundant in *RcAP2/ERF* promoters, thus implying that *RcAP2/ERFs* may be regulated by other TFs. A relatively large number of *cis*-regulatory elements share homology with TFs and mediate hormonal signaling or abiotic stress tolerance. This indicates that *RcAP2/ERF* TF regulation is integral to the growth and stress responses of the rose.

DREB proteins comprise one of the largest group of AP2/ERF TFs and are widely detected in several plants, such as rice (*Oryza satviva*) OsDREB2A [54], soybean (*Glycine max*) GmDREB2 [55], maize (*Zea mays*) *ZmDREB2.7* [56], tomato (*Solanum lycopersicum*) *SlDREB2* [57], as well as some desert plants, such as *Eremosparton songoricum* DREB2B [58], *Caragana korshinskiis* DREB1 [59] and *Tamarix hispida* DREB [60]. These DREB proteins are known to play key roles in response to abiotic stresses, particularly for A1 and A2 DREB subgroup members. In the present study, we chose an DREB A2 subgroup member, *RchiOBHmChr2g0160621,* and named it RcDREB2B, which has high homology with AtDREB2A and AtDREB2B (Fig. 5a). Both of these two genes are induced by dehydration and high-salt stress, and *AtDREB2A* is one of the most common TF in regulating drought-responsive gene expression through regulating stress-inducible genes that contain DRE sequences [7]. Our results showed that *RcDREB2B* expression was significantly suppressed, especially for MD in the root samples, under drought stress treatments, (Fig. 5b). The expression pattern of *RcDREB2B* in response to drought stress does not corroborate with other reported DREB2B members, suggesting the possible occurrence of diverse functions among different plant species. Also, the regulation analysis conducted for the expression of *RcDREB2B* under the treatments we tested, should further be investigated.

Many studies have reported that DREB gene products being involved in abiotic stress responses and enhancement of plant tolerance towards abiotic stress due to overexpression of DREB genes [5]. The current study shows that overexpression of *RcDREB2B* in *Arabidopsis* resulted in enhanced sensitivity to high salt, ABA, PEG at the germination and seedling stage, together with a less increment in root length. Similar results have been observed with transgenic rice overexpressing physic nut (*Jatropha curcas*) *JcDREB2* gene [61], which caused increased sensitivity to salt stress. Until now, the mechanism regulating the function of DREB2-type genes under abiotic stress is not well-studied. 26S proteasome often degrades DREB2 protein stability under non-stress conditions, through its negative regulation domain [62]. AtDREB2A was ubiquitinated by DREB2A-INTERACTING PROTEINS abbreviated as DRIP1 and DRIP2 in the nucleus [62]. Ubiquitination presumably leads to its subsequent DREB2 type protein degradation, thus negatively regulating the response to drought. Detail evidence needs to be illustrated not only transcriptional regulation but post-translational modification like ubiquitination or phosphorylation, and these may be a suitable conserved mechanistic pathway for the degradation or activation of DREB2 type proteins under stress conditions.

The role of ABA in many plant processes such as dormancy and formation of seeds, growth arrest and inhibition of germination in the early seedling stage under unfavorable environmental conditions has been well established [63]. Our study suggested that *RcDREB2B* expression in *Arabidopsis* restricted seed germination and seedling growth by upregulating the expression of ABA-regulated TF *AtKAI2* (Fig. 8). This gene has been shown to play a key role in ABA inhibition during the germination of seeds and the early development of seedlings [63]. *AtKAI2* upregulation causes the ABA allergic phenotype of *RcDREB2B* during seed germination and early seedling development. This activation may have caused the ABA accumulation to increase under salt or drought conditions and caused the plants to exhibit the concurrent salt and drought sensitivity, leading to enhanced seed sensitivity. *RcDREB2B*-OE plants exhibited hypersensitivity to PEG at the germination stage, implying that RcDREB2B regulates osmotic stress tolerance via ABA-mediated cell signaling. In addition, ABF3/4 exhibited less expression level in *RcDREB2B*-OE lines than VC controls. Depressed expression levels of ABF3/4 may reflect a negative feed-back loop needed to dampen the increased ABA sensitivity during germination and post-germination stage in *RcDREB2B* transgenic plants. These results are consistent with previous studies on some DREB TFs, which possess the ability to be activated by ABA and mediate their downstream gene expression enabling a plant to survive any stressful environment.

## Conclusions

To summarize, in our investigation, we identified 135 AP2/ERF TFs in the rose genome. Their classification and evolutionary relationships were elaborated using a phylogenic, conserved motif, and gene structure analysis. *Cis*-acting elements of these AP2/ERF TFs revealed that these genes mediate development and stress responses in rose plants. The AP2/ERF family bioinformatics analysis results provide primary resources to investigate the molecular regulation of rose species. Besides, we characterized a novel rose AP2/ERF gene *RcDREB2B*, the expression of which was repressed by drought. *RcDREB2B*-overexpressing transgenic *Arabidopsis* enhanced sensitivity to salt and drought stress and exhibited an ABA-hypersensitive phenotype. The outcome of this investigation will help to increase our understanding of the roles played by RcDREB2B in a rose in responses to abiotic stress.

## Methods

### Sequence identification and examination of *Rosa chinensis RcAP2/ERF* genes

We conducted a Hidden Markov Model (HMM) through Pfam’s (http://pfam.sanger.ac.uk) RcAP2/ERF amino acid sequences [40] to check whether an AP2 domain (PF00847) was present in the Rosaceae database of genomes (https:/www.roseceae.org/). Amino acid composition, chemical, and physical characteristics of *RcAP2/ERF* TFs were examined using the ProtParam method (https://web.expasy.org/protparam/) [64]. WoLF PSORT (https://wolfpsort.hgc.jp/) [65] was used to make predictions regarding the subcellular localization of proteins inside cells.

### Gene structure, chromosomal localizations, conserved motif, phylogenic, and promoter analyses

The default settings of ClustalW facilitated the investigation of the *RcAP2/ERF* structure [66]. The Gene Display Structure Server (http://gsds.gao-lab.org/) was used to evaluate the components of the *RcAP2/ERF* introns and exons. The MEME tool (http://meme-suite.org/tools/meme) was employed to predict and analyze the motifs of *RcAP2/ERF* proteins, with parameters set as follows: motif width 6–200, the maximum number of motifs: 10 and default values were used for the remaining parameters [67]. RcAP2/ERF TFs were mapped to rose chromosomes according to the *RcAP2/ERF* location presented by Mapchart v2.2 software and the Circos tool [68]. AP2/ERF proteins of apple (*Malus domestica*) and *Arabidopsis* were obtained from the plant transcription factor database (http://plntfdb.bio.uni-potsdam.de/v3.0/). The phylogenetic relationship of RcAP2/ERF was established by the neighbor-joining method with 1000 bootstraps values and was then visualized with the help of MEGA 7.0 software [69]. The evolutionary background was derived using an online tool of Evolview [70]. *RcAP2/ERF* genes promoter regions, the 1.5 kb sequence upstream of initiation codons of each gene were analyzed and annotated in Plant *cis*-acting Regulatory DNA Elements (PlantCARE) [71].

### Plant growth and drought stress treatment

Rose (*Rosa chinensis* ‘Mutabilis’) plant seeds were harvested at Qingdao Agricultural University trail garden where the annual average temperature was 12.7 °C, however, the annual average sunshine hours were 2622.3 h. After 2 months’ verification, planter boxed seedlings grew in media (peat/vermiculite = 1:1) for 45 days at 23 ± 1 °C under a photoperiod comprising 16 h of light and 8 h of dark. Then, the seedlings were exposed to drought stress for mild drought stress (MD) for 5 days and severe drought stress (SD) for 10 days. The controls were maintained with water irrigation under the same growth conditions. Three plants were included in each treatment and the leaves and roots were collected at the indicated time points with three biological replicates.

### RNA isolation and quantitative reverse transcription PCR analysis

Total RNA was extracted from rose leaves and roots utilizing the RNA Easy Fast Kit (Tiangen, Beijing, China). Sample RNA in *Arabidopsis* isolated from leaves of 3-week-old seedlings from the *RcDREB2B* transgenic lines (OE #4, OE #5, and OE #16) and control (VC) plants. After the validation of RNA concentrations using Nanodrop™2000 (Thermo Fisher Scientific, USA), first-strand cDNA synthesis with 1 μg RNA was performed using the PrimeScript first-strand cDNA synthesis equipment (TaKaRa, Dalian, China).

For evaluating the rate of gene transcription, RT-qPCR was conducted with SYBR Premix Ex Taq (TaKaRa, Dalian, China). A reaction volume of 20 μl was used which comprised: RNase-free water (8.2 μl), template (with 5-fold dilutions of cDNA) (1 μl), each primer (0.4 μl), and 2 × SYBR Premix (10 μl). The RT-qPCR protocol involved: 30 s at 95 °C; 95 °C for 40 × 5 s cycles and 34 s at 60 °C with a concluding step of 15 s at 95 °C. *RcUBI1* and *AtACTIN2* were used as internal controls in rose and *Arabidopsis*, respectively. Calculation of the expression level was conducted, using 2^-ΔΔt^ as described by Livak and Schmittgen [72]. Three separate replicates were carried out for RT-qPCR. Primers are listed in Table S2.

### Amplification and sequence analysis of *RcDREB2B*

The amplification of *RcDREB2B* primers was designed according to the full-length coding sequence of *RchiOBHmChr2g0160621* and was used to amplify the cDNA sequence in rose leaves under MD treatment. For sequencing, the products were purified and cloned into the pMD18-T vector (TakaRa, Dalian, China). The molecular weight and isoelectric point (pI) of RcDREB2B were estimated with ExPASy (http://expasy.org/tools/pi_tool.html) for computation. DNAMAN software was used to perform multiple sequence alignment. The neighbor-joining method was used to generate phylogenetic analysis and a display was made using Evolview [70] and MEGA7.0 [69] in the light of the similarity of amino acid sequences.

### Subcellular localization of RcDREB2B

Overlapping the PCR method was used to link the open reading frame (ORF) of RcDREB2B without the stop codon, and *Sal* I/*Spel* I was used to digesting the recombinant fragment which was then inserted into the corresponding sites of the pCAMBIA 1300-GFP vector [24] to generate pCAMBIA 1300-RcDREB2B-GFP. After verification by sequencing, the construct of pCAMBIA 1300-RcDREB2B-GFP and pCAMBIA 1300-GFP control was converted into *Arabidopsis* (Columbia) mesophyll protoplast using the PEG-calcium transfection method [73]. A laser scanning confocal microscope (TCS SP5, Leica, Germany) was employed to observe green fluorescent protein signals of the protoplast cells.

### Transactivation analysis in yeast

The pGBKT7-RcDREB2B construct was generated by inserting the full-length sequence of RcDREB2B into the *Nde* I and *Eco*R I sites of pGBKT7. The pGAL4 plasmid (positive control), pGBKT7 vector (negative control), and pGBKT7-RcDREB2B recombinant plasmid were then transmitted into Y2H Gold, the yeast strain that had the reporter genes *His3* and *LacZ*. Transformed cells were verified using PCR followed by plating on SD/−Trp or SD/−Trp-His-Ade medium. In the following step, the plates were incubated at 30 °C for 3 d, and the resulting clones were used in a 5-bromo-4-chloro-3-indolylb-D-galacto-pyranoside (X-gal) assay to examine the transactivation ability of RcDREB2B.

### Seed germination, root growth, and superoxide assay

Using the *Agrobacterium tumefaciens* GV3101 harboring pCAMBIA 1300-RcDREB2B, wild-type (WT) *Arabidopsis* (Columbia) was transformed via the floral dipping method [74]. Transgenic *Arabidopsis* was selected on Murashige and Skoog (MS) medium containing 50 mg/L hygromycin and confirmed by using PCR analysis. The T3 homozygous positive lines were employed for all further experiments. Vector plants (VC) or transgenic *Arabidopsis* seeds were sown on MS and plants were grown at 23 ± 1 °C under a 16-h light/8-h dark cycle.

For germination assays, *Arabidopsis* seeds were surface-sterilized with 75% alcohol for 5 min and 2% NaClO for 10 min, respectively. To determine the effects of NaCl, ABA, or PEG on seed germination, seeds from VC and T3 transgenic plants (OE#4, OE#5, and OE#16) were transferred to MS agar plates or MS agar plates saturated with various concentrations of NaCl (0, 50, 100,150 and 200 mM), ABA (0, 1.2, and 1.6 μM) or PEG 4000 (0, 4, 8, 12 and 16%), respectively. Then, seeds were incubated at 4 °C for 72 h and were transferred to a growth chamber (16 h light / 8 h dark photoperiod, approximately 100 μmol m^− 2^ s^− 1^ photosynthetic photon flux) at 23 ± 1 °C for germination. After 9 d, the rates of seed germination were recorded. The emerging of radical and greening of cotyledon was considered seed germination.

To determine the growth of root, 6 d old seedlings were carefully transferred to MS media containing different concentrations of NaCl (0, 100, 150, or 200 mM) or ABA (0 or 100 μM) and incubated for 10 d. The position of the root tips was marked at the time of transfer, and the root growth was measured from this mark after 7 d. The root elongation and lateral root number were analyzed by Image J (http://rsb.info.nih.gov/ij). Three independent biological replicates were conducted for salt or ABA experiments.

Nitro blue tetrazolium (NBT) and hydrogen peroxide by 3,3-diaminobenzidine (DAB) staining were carried out for situ detection of superoxide as described previously [75]. Following 10 d of growth, H_2_O_2_, and O_2_^−^ were found in *RcDREB2B* transgenic and VC seedlings when immersed for 1 h in either water (CK) or 200 mM NaCl. Concentrated and 90% ethanol was then used for treating the seedlings for destaining before photographing with a Stemi DV4 light microscope (Carl Zeiss, Gottingen, Germany). Ten technical and three biological replicates were used for each tested condition. Mean significant differences were compared by ANOVA analysis at *P* ≤ 0.05 utilizing SPSS 24.0 software (IBM, Armonk, NY, USA).

### Supplementary Information

**Additional file 1: Table S1.** Information of gene ID, physical position, and properties of RcAP2/ERF proteins.

**Additional file 2: Table S2.** Primer sequences used in this study.

**Additional file 3: Figure S1.** Multiple sequence alignment between RcDREB2B and other plant DREB proteins. The conserved DREB AP2/ERF domain is seen as the underlined segment. Stars denote the amino acid residues in the AP2/ERF domain that have been reported to be conserved. NLS and AP2-domain are marked with red and blue solid boxes, respectively. The underlined asterisks of red, blue, and black indicate YRG, WLG, and RAYD motif, respectively.

**Additional file 4: Figure S2.** Morphological phenotypes of VC and *RcDREB2B* transgenic *Arabidopsis*. (A) Flowers morphological of VC and *RcDREB2B* transgenic lines. Bar = 1 cm. (B) The flower diameter, petal length, petal width, and single petal area of VC and *RcDREB2B* transgenic lines. 3-week-old plants were photographed and the total areas, petal length, and width were determined by using Image J software. Error bars indicate SE (*n* = 3).

**Additional file 5: Figure S3.** Quantities analysis of H_2_O_2_ (A) and O_2_^−^ (B) in VC and *RcDREB2B* transgenic *Arabidopsis*.

**Additional file 6: Figure S4.** Germination analysis of VC and *RcDREB2B* overexpressing lines in response to PEG. (A) Germination of VC and three *RcDREB2B* transgenic lines on MS plus different concentrations of PEG 4000 (0, 4, 8, 12 and 16%) after 9 days. (B) Statistical analyses of germination rates indicated in (A).

**Additional file 7: Figure S5.** The whole gels for each of the cropped gel images shown in Fig. 6a of the main text.

## Data Availability

The RcDREB2B sequence data was deposited in the NCBI GenBank under accession number MH152409.

## Abbreviations

TF: Transcription factor
AP2/ERF: APETALA2/ethylene-responsive factor
NLS: Nuclear localization signal
RT-qPCR: Real-time quantitative PCR
ORF: Open reading frame
MS: Murashige and Skoog
NBT: Nitro blue tetrazolium
DAB: 3,3-diaminobenzidine
