# Correction: Whole-genome characterization of *Rosa chinensis* AP2/ERF transcription factors and analysis of negative regulator *RcDREB2B* in Arabidopsis

**DOI:** 10.1186/s12864-024-10561-2

**Published:** 2024-07-01

**Authors:** Wei Li, Ziwen Geng, Cuiping Zhang, Kuiling Wang, Xinqiang Jiang

**Affiliations:** College of Landscape Architecture and Forestry, Qingdao Agricultural University, Qingdao, 266000 China

**Correction**: *BMC Genomics***22**, 90 (2021). 10.1186/s12864-021-07396-6

Following publication of the original article it was reported that there was an error in Fig. 7. The same photo was used for OE#16 under 1.2 µM ABA and VC under 1.6 µM ABA.

The correct Fig. 7 is given in this Correction article.

**Fig. 7:**
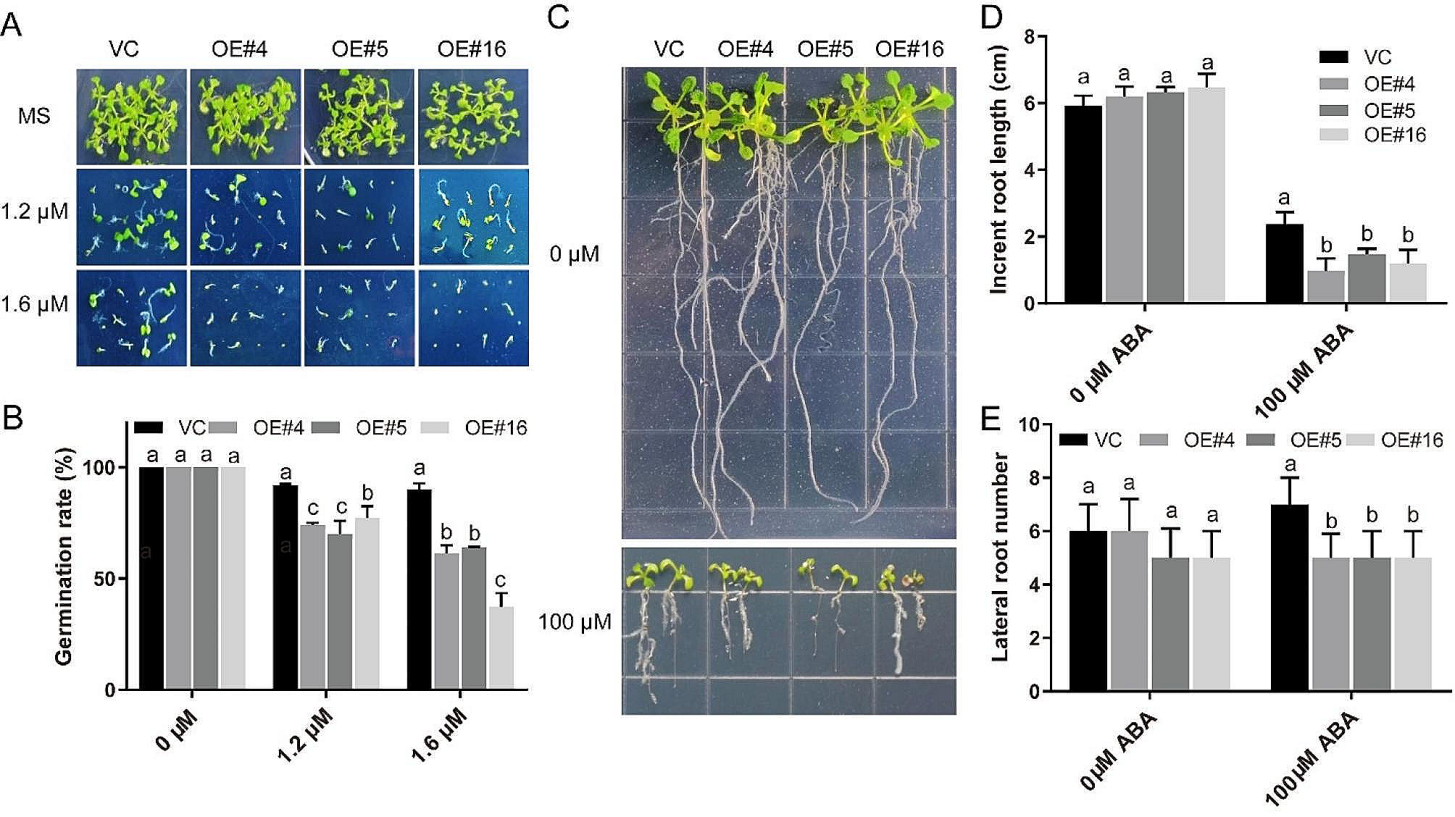
Germination and root growth analysis of VC and *RcDREB2B* overexpressed lines in response to ABA. **a** Seed germination performance of VC and *RcDREB2B* overexpressed lines on MS with different ABA concentrations (0, 1.2 µM, 1.6 µM) after 9 days. **b** Statistical analyses of germination rates are indicated in (**a**). Seed germination rates were measured after 9 d growth. **c** Root growth of VC and transgenic seedlings on MS agar plates containing 0 µM or 100 µM ABA. Representative photos were taken following 10 days of the transfer. Seedlings were 6-days-old at the time of transfer. Increment in root length (**d**), and lateral root number (**e**) of VC and *RcDREB2B* transgenic plants in response to ABA. Different letters indicate significant differences in three independent experiments at *P* < 0.05 using one-way ANOVA analysis

