# Supplementary figures and images for: Whole-genome characterization of Rosa chinensis AP2/ERF transcription factors and analysis of negative regulator RcDREB2B in Arabidopsis
Source: BMC Genomics. 2021 Jan 28;22:90. doi: 10.1186/s12864-021-07396-6 (PMC7844920; doi:10.1186/s12864-021-07396-6)

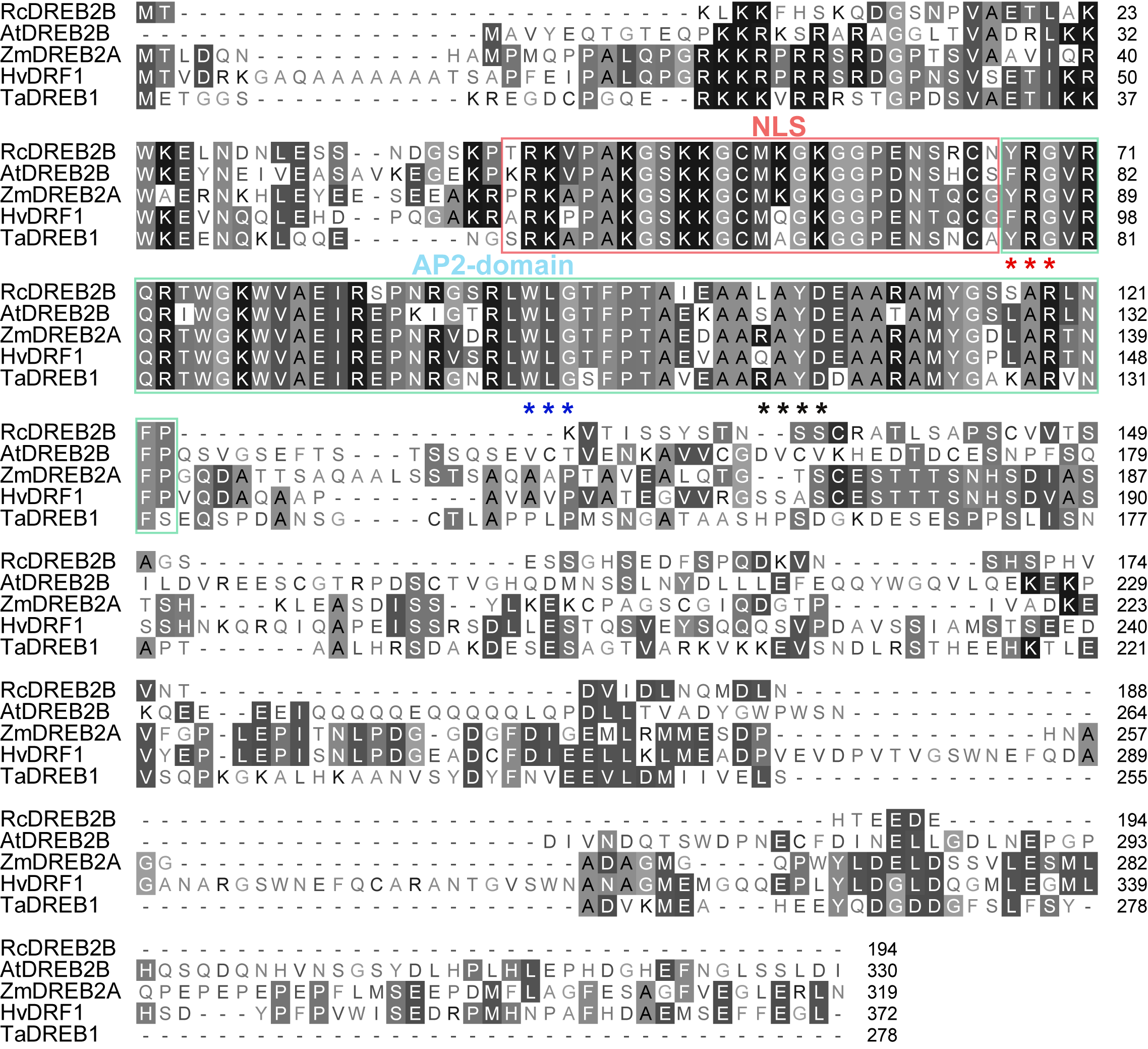

Supplement: Supplementary file 3 — Additional file 3: Figure S1. Multiple sequence alignment between RcDREB2B and other plant DREB proteins. The conserved DREB AP2/ERF domain is seen as the underlined segment. Stars denote the amino acid residues in the AP2/ERF domain that have been reported to be conserved. NLS and AP2-domain are marked with red and blue solid boxes, respectively. The underlined asterisks of red, blue, and black indicate YRG, WLG, and RAYD motif, respectively. [file 12864_2021_7396_MOESM3_ESM.tif]

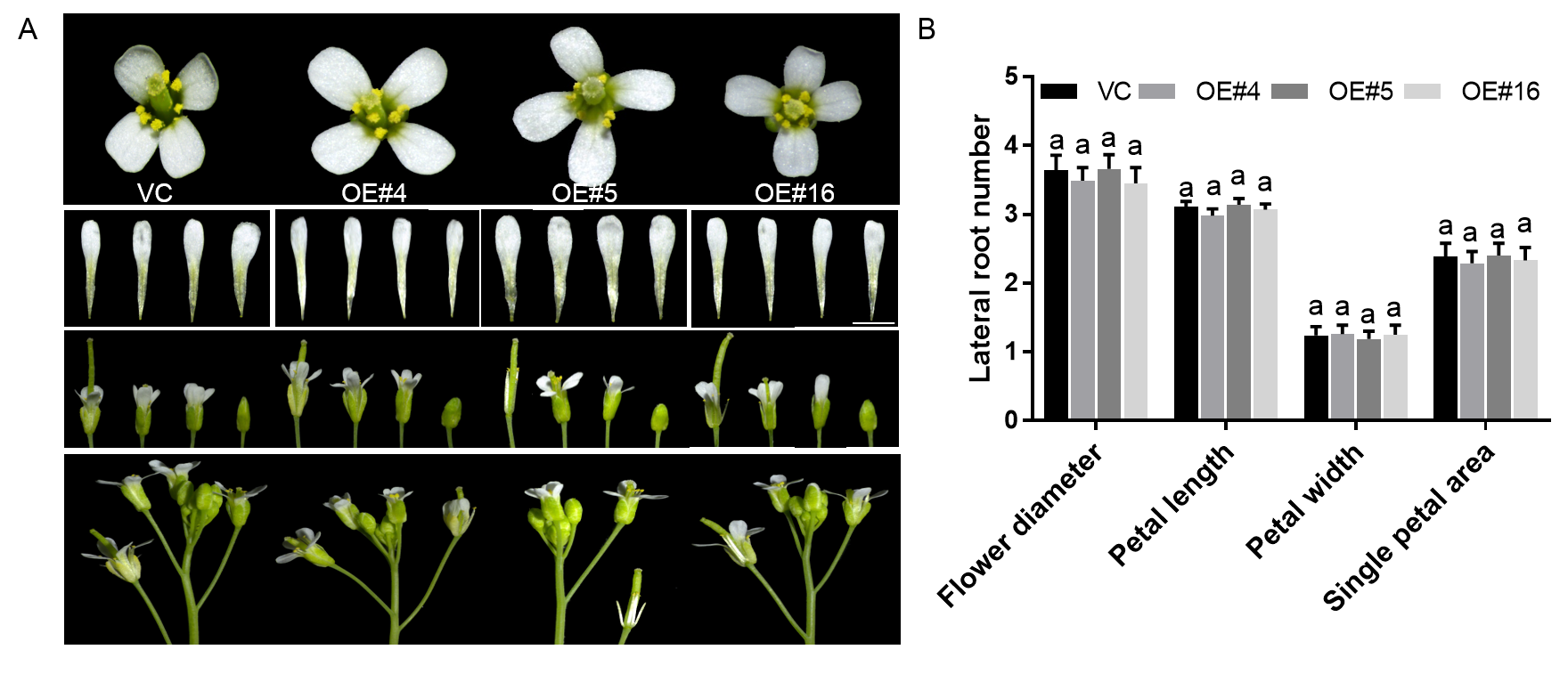

Supplement: Supplementary file 4 — Additional file 4: Figure S2. Morphological phenotypes of VC and RcDREB2B transgenic Arabidopsis. (A) Flowers morphological of VC and RcDREB2B transgenic lines. Bar = 1 cm. (B) The flower diameter, petal length, petal width, and single petal area of VC and RcDREB2B transgenic lines. 3-week-old plants were photographed and the total areas, petal length, and width were determined by using Image J software. Error bars indicate SE (n = 3). [file 12864_2021_7396_MOESM4_ESM.tif]

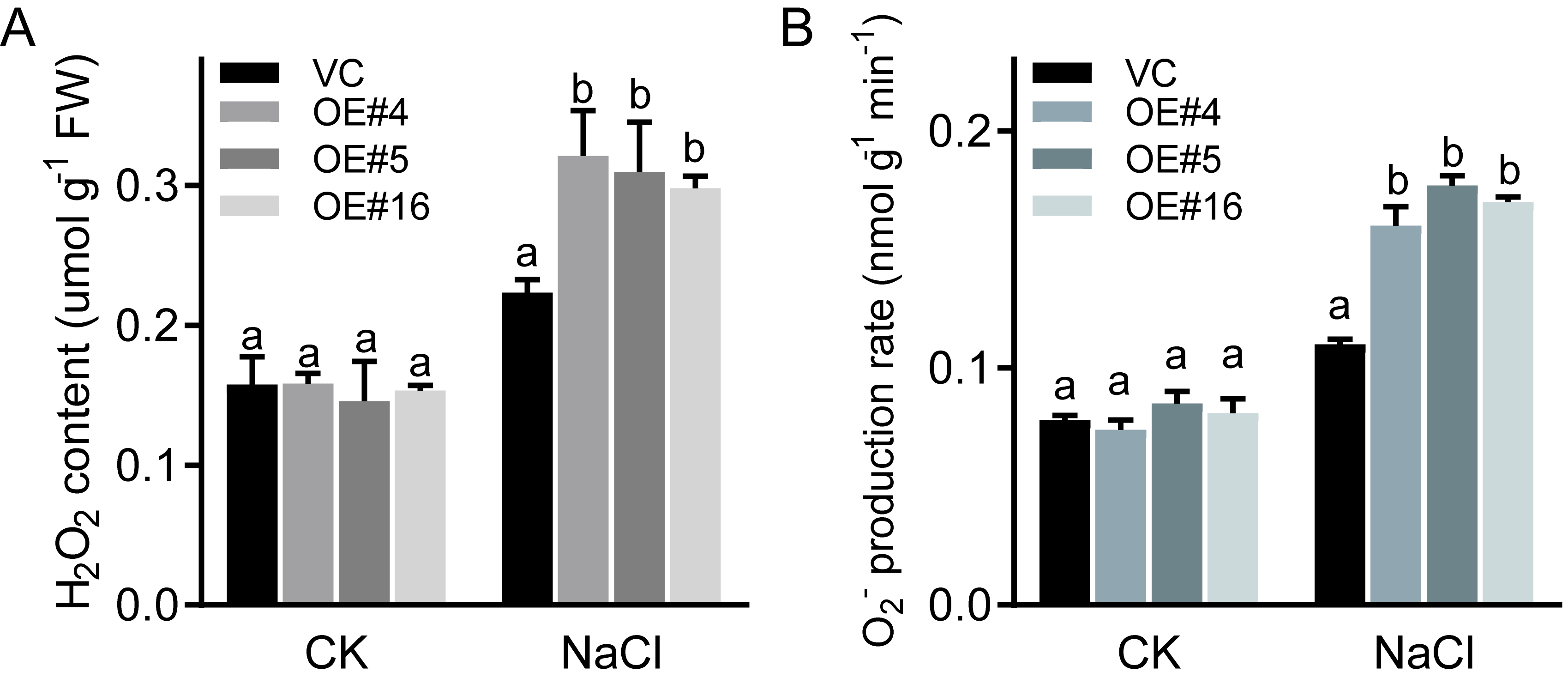

Supplement: Supplementary file 5 — Additional file 5: Figure S3. Quantities analysis of H2O2 (A) and O2− (B) in VC and RcDREB2B transgenic Arabidopsis. [file 12864_2021_7396_MOESM5_ESM.tif]

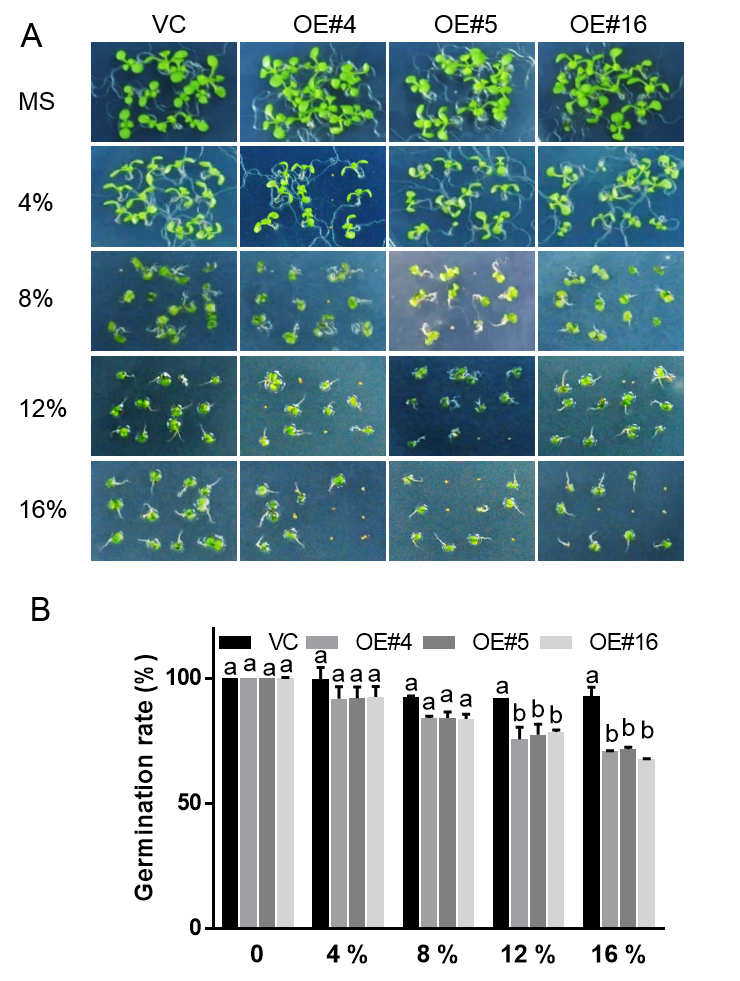

Supplement: Supplementary file 6 — Additional file 6: Figure S4. Germination analysis of VC and RcDREB2B overexpressing lines in response to PEG. (A) Germination of VC and three RcDREB2B transgenic lines on MS plus different concentrations of PEG 4000 (0, 4, 8, 12 and 16%) after 9 days. (B) Statistical analyses of germination rates indicated in (A). [file 12864_2021_7396_MOESM6_ESM.tif]

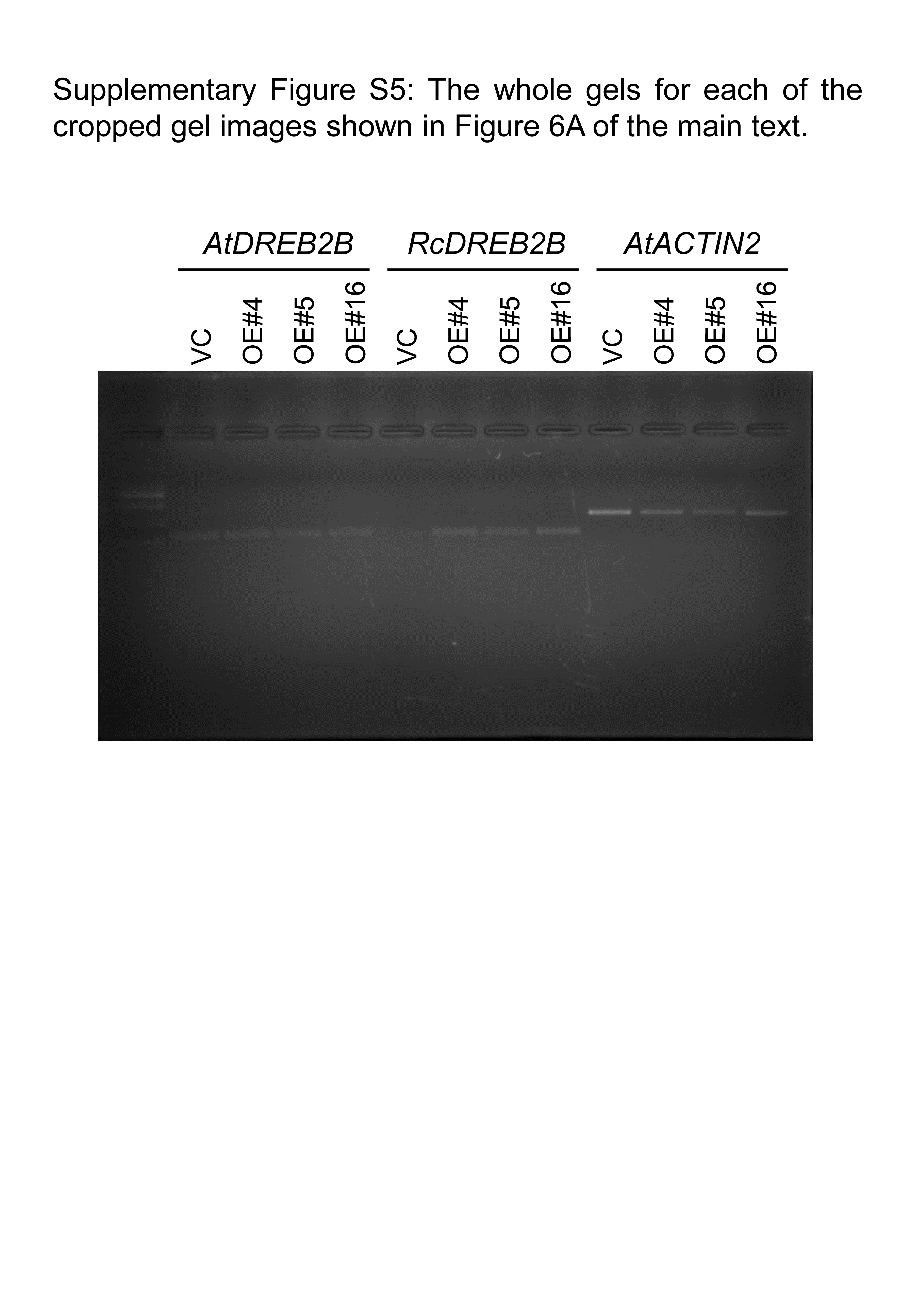

Supplement: Supplementary file 7 — Additional file 7: Figure S5. The whole gels for each of the cropped gel images shown in Fig. 6a of the main text. [file 12864_2021_7396_MOESM7_ESM.tif]
